# Studies of Bis-(Sodium-Sulfopropyl)-Disulfide and 3-Mercapto-1-Propanesulfonate on/into the Copper Electrodeposited Layer by Time-of-Flight Secondary-Ion Mass Spectrometry

**DOI:** 10.3390/molecules27238116

**Published:** 2022-11-22

**Authors:** Robert Mroczka, Agnieszka Słodkowska, Agata Ładniak

**Affiliations:** Laboratory of X-ray Optics, Department of Chemistry, Institute of Biological Sciences, Faculty of Medicine, The John Paul II Catholic University of Lublin, Konstantynów 1J, 20-708 Lublin, Poland

**Keywords:** copper electrodeposition, SPS (bis-(sodium-sulfopropyl)-disulfide), MPS (3-mercapto-1-propanesulfonate), TOF-SIMS, cyclic voltammetry

## Abstract

Interactions of functional additives SPS (bis-(sodium-sulfopropyl)-disulfide), MPS (3-Mercapto-1-Propanesulfonate), and Cl accumulated and incorporated on/into a copper electrodeposited layer were studied using time-of-flight secondary-ion mass spectrometry (TOF-SIMS) in combination with cyclic voltammetry measurements (CV). It was shown that the Cl and MPS surface coverage is dependent on the applied overpotential and concentration of Cl, SPS, or MPS in the solution. Detailed discussion on the mechanism of yielding CH_2_SO_3_^−^, C_3_H_5_SO_3_^−^, CuSC_3_H_6_SO_3_^−^, and CuS^−^ fragments and their assignment to the gauche or trans conformation was proposed. The mechanism of the process of incorporation and re-adsorption of MPS on/into a copper surface under electrochemical conditions without and with chloride ions and its impact on electrochemical properties was proposed. Moreover, it was shown that the presence of chloride ions, the ratio gauche/trans of MPS molecules, as well as the ratio chloride/thiols demonstrate a high impact on the accelerating abilities. Comparative studies conducted under open circuit potential conditions on the nitinol and copper substrate allowed for the identification of specific reactions/interactions of MPS, or SPS and Cl ions on the nitinol and copper surface.

## 1. Introduction

Chemistry of MPS and SPS playing a primary role as additives for copper electrodeposition processes has been thoroughly studied [1,2,3]. SPS dissociates on the copper surface to its two monomeric forms (MPS). Without the presence of other additives (chloride, polyethylene glycol) MPS-adsorbed molecules on the copper surface demonstrate minor inhibiting properties by playing the role of a blocking barrier for the reduction of Cu^2+^ ions to Cu [4,5]. In the presence of chloride ions, both MPS and SPS change properties from inhibiting to accelerating [5,6]. It has been claimed by some authors [1,7] that the formation of very stable complex Cu(I)ClMPS^−^ is responsible for the acceleration of copper deposition. Some more recent studies [8] showed that sulfonate groups help to partially remove the hydration shell of Cu^2+^ that accelerates the reduction of Cu^2+^ to Cu^+^ and transfer the latter species to a chloride adlayer. In that way, SPS or MPS acts as an efficient factor for the desolvation step while chloride ions are important for forming Cu–Cl complexes that are reduced to Cu by single electron transfer.

However, electrochemical techniques such as cyclic voltammetry supply only indirect evidence for the existence of numerous possible complexes and adsorbents on the electrode or near-surface of the electrode [1]. Moreover, it was postulated [9] that cyclic curves and the extracted Tafel slopes cannot be unequivocal tools for determination- of mechanistic model of kinetics on the copper surface during electrodeposition.

Due to this reason, numerous investigations of physicochemical interactions between components of the electrochemical bath under an open circuit potential regime as well as under electrochemical conditions using an additional complementary technique for evaluation of the electrode surface and electroplating bath chemistry were developed. For example, the mechanism of copper electrodeposition and accumulation of additives onto copper deposit by in situ studies by means of combining scanning transmission microscopy with electrochemical scanning tunnelling microscopy EC-STM [10,11,12,13] or combined sum frequency generation (SFG) spectroscopy with scanning tunnelling microscopy (STM) [14] were conducted.

Molecular and elemental characterisation of compositions and its by-products in the electroplating bath were provided by mass spectroscopy [15,16,17], liquid chromatography mass spectrometry (LC-MS) [18], and high-pressure liquid chromatography (HPLC) [19].

It is well known that in processes carried out under electrochemical conditions the additives (SPS or MPS) at low concentrations in the presence of chloride ions play the accelerating role [20,21].

Postulated chemical reactions under electrochemical conditions were conducted under non-electrochemical conditions and were studied by X-ray diffractometry (XRD) [22], nuclear magnetic resonance (NMR) [23,24], UV-VIS spectrometry [21,25], and inductively coupled plasma mass spectrometry (ICP-MS) [13]. Moreover, molecular identifications of additives accumulated on the electrodeposited copper layers were studied using spectroscopic techniques: Fourier transform infrared (FTIR) [3], surface-enhanced Raman spectroscopy (SERS) [3,20,26], X-ray photoelectron spectroscopy (XPS) [27], combined laser ablation/ionization with secondary-ion mass spectrometry (LIMS/SIMS) [28], glow discharge optical emission spectroscopy (GD-OES) [29], as well as time-of-flight secondary-ion mass spectrometry (TOF-SIMS) [29,30,31,32,33,34,35]. Ex situ studies of additives and base components in the solution by means of electron paramagnetic resonance (EPR) [5] and electrospray ionization mass spectrometry (ESI-MS) [3] were also carried out. Quantum chemistry calculations as complementary and supporting studies to experimental techniques based on density functional theory (DFT) were also applied [3,12,13,20,26].

Among available in situ methods of surface analysis, surface-enhanced Raman spectroscopy [3,8,20] allows for the identification of the conformation of gauche and trans thiolate molecules adsorbed on the copper surface during electrodeposition. However, for many years the special preparation of copper substrate before in situ measurements was served to reduce a problem with reproducibility. To overcome that difficulty, shell-isolated nanoparticle-enhanced Raman spectroscopy (SHINERS) combined with cyclic voltammetry was developed and applied [20,36,37,38,39,40]. However, despite significant benefits of the latter modification of the SERS technique, it still possesses some limitations that do not allow for its application to real-world samples for application in industry. For example, in order to maintain the shape of Au/SiO_2_ nanospheres, very low amounts of copper have to be electrodeposited, which limits investigations on low concentrations copper sulphate in the solution. Moreover, a relatively low thiolate detection level during in situ measurements by means of SHINERS determines the higher, non-practical MPS or SPS concentrations. For example, in latter SHINERS studies [8] the SPS and copper sulphate concentrations were equal to 1 mM (155 ppm) and 10 mM, respectively. Moreover, due to a relatively long acquisition time required for stabilisation of the Raman signal, routinely used linear speed voltammetry cannot be applied while staircase voltammetry is used as alternative [8]. Similar limitations exist in molecular imaging methods such as STM where concentration of SPS was equal to 1 mM and solution did not contain copper sulphate [25]. Moreover, SHINERS and scanning tunnelling microscope (STM) techniques do not provide the data on chemical bonds in greater fragments of molecules that can be received from mass spectrometry.

Furthermore, SHINERS and STM methods were not applied to examine thiolate surface coverage due to unclear quantification of Raman spectra peak intensities into quantities of additives adsorbed on the surface and possible incorporation of thiolate fragments into copper deposits. Schmitt at al. [8] proposed the model of accelerating abilities of SPS/MPS molecules in the presence of chloride ions that based on the interaction of sulfonate end of MPS molecules in gauche conformation with Cu^2+^ as a process that facilitates dehydration and transfer of Cu^2+^ to the chloride adlayer. On the other hand, Hai et al. [12] proposed a model that highlights the role of chloride adlayer defects and re-adsorption cycle of thiolate molecules onto the copper surface and its impact on copper accelerating abilities. Both models do not take into account the role of possible incorporation of thiolate molecules on accelerating abilities.

Recently, we showed that static TOF-SIMS can be successfully applied in molecular studies of polyethylene glycol in the presence of chloride ions during copper electrodeposition [34]. Since the TOF-SIMS method cannot be used for in situ measurements, we designed and applied an experiment that allowed us to evaluate the chemistry of the copper surface as a function of applied potential. During CV experimentation, nitinol wire served as a working electrode and was withdrawn at a constant speed. In this manner, the exact positions along the wire correspond to the applied overpotential during CV experimentation. Moreover, due to high concentration of copper sulphate in solution, the method of rinsing samples after CV experiment was optimised to avoid one side from salting out of copper sulphate, and on the other side to preserve a maximum amount of adsorbate material on the surface. More details can be found in our previous paper [34] and in the experimental section.

From this point of view, serving the TOF-SIMS method for identification of small molecules such as thiolate that can form strong chemical bonds on the copper surface seems to be very useful. In our previous paper [35] we identified the most characteristic fragments of thiolate on the copper surface electrodeposited under galvanostatic conditions. In the present work, we expand our investigations and focus our efforts on areas unexplored by the TOF-SIMS in the following aspects: (1) the evaluation of the most relevant reactions between additives and cupric/cuprous ions on the copper surface under open circuit potential and reactive condition; (2) the trial of resolving the problem of identification of gauche/trans conformation of the thiolate in the TOF-SIMS mass spectra; (3) explanation of the possible incorporation of thiolate and chloride ions into copper deposit during electrodeposition; (4) determination of the role and impact of additives conformation, their possible incorporation into copper surface, as well surface coverage on the accelerating abilities; (5) evaluation, unification and application of proposed literature model of interactions between additives [8,12,41] for industry applied copper electroplating bath.

## 2. Results and Discussion

### 2.1. Dip-Coating (DC) Experiments on the Bare Nitinol Wire and Pre-Plated Nitinol Wire—Evaluation of Chemistry MPS and SPS under Non-Electrochemical Condition

**Figure 1:**
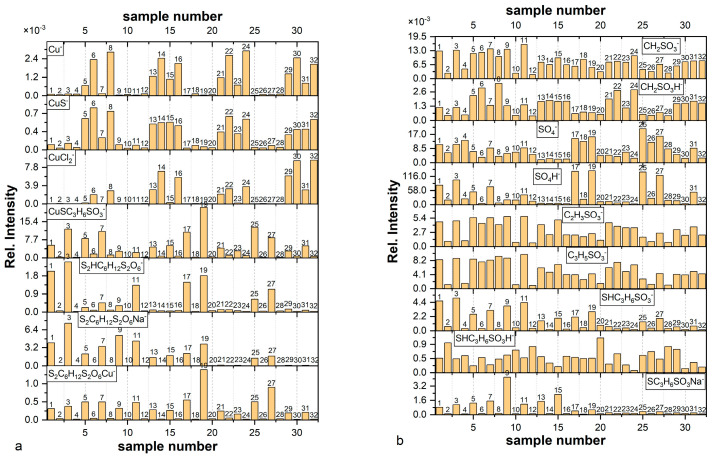
(**a**) Distribution of relative intensity Cu^−^, CuS^−^, CuCl_2_^−^, CuSC_3_H_6_SO_3_^−^, S_2_HC_6_H_12_S_2_O_6_^−^, S_2_C_6_H_12_S_2_O_6_Na^−^, and S_2_C_6_H_12_S_2_O_6_Cu^−^ identified in the TOF-SIMS mass spectra for 32 samples prepared in dip-coating experiment. (**b**) Distribution of relative intensity CH_2_SO_3_^−^, CH_2_SO_3_H^−^, SO_4_^−^, SO_4_H^−^, C_2_H_3_SO_3_^−^, C_3_H_5_SO_3_^−^, SHC_3_H_6_SO_3_^−^, SHC_3_H_6_SO_3_H^−^, and SC_3_H_6_SO_3_Na^−^ identified in the TOF-SIMS mass spectra for 32 samples prepared in dip-coating experiment.

Figure 1a,b shows distribution of the most prominent fragments identified in the TOF-SIMS spectra. The *m/z* ratios and assignments of selected fragments are included in Table 1.

The SO_3_^−^ fragment can be yielded from sulfonate ends of thiolate molecules as well as from sulphate ions. SO_4_^−^ and HSO_4_^−^ correspond to sulphate ions. CH_2_SO_3_^−^, C_2_H_3_SO_3_^−^, C_3_H_5_SO_3_^−^ are yielded from complexated and uncomplexated forms of thiolate.

The fragment CH_2_SO_3_^−^ is yielded through homolytic cleavage C–C bond of terminal sulfonate end whilst the possible fragmentation pathways that lead to fragments: C_2_H_3_SO_3_^−^, C_3_H_5_SO_3_^−^ are as follows:

CuS(CH_2_)_3_SO_3_ → Cu^•^ + ^•^S(CH_2_)_3_SO_3_^−^ → S^−^ + ^•^CH_2_CH_2_CH_2_SO_3_^−^ → SH^•−^ + ^•^CH_2_CH^•^CH_2_SO_3_ → SH^•−^ + CH_2_ = CHCH_2_SO_3_^−^ (1)

^•^S(CH_2_)_3_SO_3_^−^ → S^−^ + ^•^CH_2_^•^ + ^•^CH_2_CH_2_SO_3_^−^ → SH^•−^ + ^•^CH_2_^•^ + ^•^CH_2_CH^•^SO_3_^−^ → CH_2_ = CHSO_3_^−^ (2)

For all nitinol substrates the intensity of Cu^−^ fragment is negligible in comparison to the copper pre-plated nitinol wires (Figure 1a,b). It unambiguously means that the Cu^−^ fragment is mainly yielded from the copper substrate. Low intensity of Cu^−^ for samples with copper substrate is determined by the thicker thiolate or thiolate/Cl layer. For example, the thickest layer among layers deposited on the copper substrate is observed for Sample 7 (copper substrate immersed in MPS solution for 60 s). On the other hand, Sample 8 prepared in the same way but demonstrates high intensity of Cu^−^ after rinsing because only a thin layer, very likely monolayer of MPS, remains on the substrate. We can observe similar behaviour for all unrinsed/rinsed copper samples: 5/6, 7/8, 13/14, 15/16, 21/22, 23/24, 29/30, and 31/32.

Similarly, a high intensity of CuS^−^ fragment was identified for the samples containing thiolate molecules adsorbed on the copper substrate (Samples 5–8, 13–16, 21–24, 29–32). It can strongly suggest that the CuS^−^ fragment can be assigned to Cu–S and proves the existence of the bond between S from thiolate group of MPS and metallic copper of the substrate. On the other hand, low intensity CuS^−^ fragment was also identified for layers deposited on the nitinol wire (Samples 1–4, 9–12, 17–20, 25–28). As for the TOF-SIMS spectra, it is not possible directly to distinguish the oxidation state of copper atom; this finding is helpful to distinguish Cu(0)-SC_3_H_6_SO_3_ and Cu(I)-SC_3_H_6_SO_3_^−^. For Samples 1–4, 9–12, 17–20, and 25–28 the CuS^−^ fragment can be unambiguously assigned to Cu(I)-S bond yielded from the Cu(I)-SC_3_H_6_SO_3_^−^ complex. The latter complex is considered as the main product of reaction Cu^2+^ with MPS and it is assumed as one of the crucial steps in the close loop cycle in the near electrode surface during copper electrodeposition [25] as it is shown in Reaction (3).

2 Cu^2+^ + 4 MPS → 2 Cu(I)MPS + SPS + 4 H^+^ (3)

Interaction of sulfonate group through oxygen localized in S–O with the copper substrate is determined by CuSO^−^ and CuSO_3_^−^ fragments. Distributions of CuSO^−^ and CuSO_3_^−^ are very similar to the Cu^−^. If a sulfonate group is in close proximity to the copper substrate, sputtered copper ions react easily with sulfonate yielding CuSO^−^ and CuSO_3_^−^. We can assume that a higher yield of CuSO_3_^−^ may be favoured for higher symmetry of SO_3_^−^, whilst for asymmetric structure in the case of greater stretch of S=O, a higher yield of CuSO^−^ may be generated. For example, the rinsing of Samples 29–31 provides a rise in intensity of CuSO^−^ and reduces the intensity of CuSO_3_^−^ simultaneously. It may suggest that after rinsing at lower thiolate coverage the lower asymmetry of the sulfonate group is determined by greater interaction of oxygen in S–O with copper. It arises from the fact that oxygen in S–O is in closer proximity to the copper surface due to higher availability of copper substrate.

The next important fragment is CuCl_2_^−^. In the first assumption it is rational to assign this fragment to the Cl layer adsorbed on the copper substrate. However, surprisingly, CuCl_2_ is detected not only for the layers deposited on copper from solution containing Cl^−^ ions (Samples 21, 22, 24, 29, 30, 31, 32), but also from solution without Cl^−^ ions (Samples 6, 8, 13, 14, 16). It is known that the TOF-SIMS technique is very sensitive to detection of chloride ions in the negative mode as demonstrated by high secondary ion fields [34,35,42]. In practice, the detection limit of elemental analysis reaches the level of 10 ppb. Due to this reason, even a trace amount of chloride impurities from the air or residual gases inside the TOF-SIMS instrument can be adsorbed on bare copper and can be easily detected in the form of CuCl_2_^−^ and Cl^−^ (not shown in Figure 1). We detected CuCl_2_^−^ fragments in the TOF-SIMS spectra even for extremely pure copper layer deposited by physical vapor deposition (magnetron sputtering) on silicon wafer (see Supplement Figure S1) due to contamination of copper surface by chloride impurities. Figure S1 shows distribution of Cu^−^- and CuCl_2_^−^-negative ions. It is clearly seen that the intensity of CuCl_2_^−^ is nearly ten times higher than the Cu^−^ fragment.

This means that the CuCl_2_^−^ fragment can be a good indicator for detection of not only copper substrate covered by chloride ions from the solution, but also for identification of the bare copper substrate. For example, we did not detect CuCl_2_^−^ for Samples 5 and 7 because the layer containing Cu(I)-MPS is sufficiently thick to cover copper substrate, although after rinsing (Sample 6, 8) a significant area of bare copper can easily adsorb Cl^−^ contamination from the air-yielding CuCl_2_^−^ fragments. A similar situation occurred for rinsed layers deposited from SPS solution (Samples 14, 16). On the other hand, the unrinsed layer for Sample 13 is too thin to protect the bare copper from contamination by chloride ions.

Identification of SPS molecules was determined by distribution of S_2_HC_6_H_12_S_2_O_6_ (*m/z* = 309.10), SPS–H fragment, the protonated form of SPS that unambiguously proves existence of a protonated form of SPS in an acidic environment. The significant number of SPS–H fragments is clearly visible only for unrinsed layers deposited on the nitinol substrate. The highest intensity of SPS was detected for layers obtained from the MPS-containing solutions (Samples 1, 3, 17, and 19) as an effect of Reaction (3) and for layers deposited from solutions containing SPS (11, 25, 27). For the layers received from solution containing SPS and Cl (25, 27), a significant quantity of SPS–H fragment was observed. Accordingly, the relative intensity for the fragment SPS–H for Sample 27 is about two times higher than for Sample 25. It shows that a 30-time increase in immersion time gives rise to a 2-time increase in the intensity (amount) of SPS–H. It may suggest that the amount of SPS–H is not linearly proportional to the immersion time, while increasing amount of SPS–H in a function of immersion time is obvious. On the other hand, for samples obtained from SPS-exclusive solution (without Cl) with a shorter immersion time (Sample 9) the amount of SPS–H was significantly reduced. In the case of the copper substrates long immersion time (Sample 7) the MPS-containing solution allowed for the acquisition of a noticeable amount of SPS, because at that time (60 s) a thick layer was deposited that prevented some parts of SPS molecules from direct contact with the copper substrate. Unlike Sample 5, SPS–H amount is significantly reduced due to a shorter immersion time. The lack of SPS–H for unrinsed samples received from SPS solutions on the copper substrates (Samples 13, 15, 29, 31) indicates that during contact with copper substrate disulphide bonds of SPS molecules are dissociatively retained. This observation is in full agreement with previous findings [12,43].

We also identified an unprotonated form of SPS in the layers. Assuming the number of SPS moles formed in Reaction (3) is equal to ½ moles of MPS for Samples 1–8 and 17–24 the solution can receive 0.1 M of SPS and 0.1 M of Cu(I)-SC_3_H_6_SO_3_^−^ in. Then, if [H^+^] is equal to 0.05 M, the number of moles in the protonated form of SPS (SPS–H) is equal to 0.05 M (if we assume that all SPS molecules can be protonated), whilst the remaining 0.05 moles of SPS remain unprotonated. The unprotonated forms of SPS were identified as the sodium and copper adducts: SPS–Na (*m/z* = 331) and SPS–Cu (*m/z* = 371) occurring in the unrinsed layers deposited on nitinol as well as copper substrate (samples with odd numbers).

The existence of the fragment with mass 371 (Cu–SPS) was previously reported by Frank et el. [19] in the solution phase identified by means of the LC-MS technique as an effect of reaction:

2 Cu^2+^ + 4 HS(CH_2_)_3_SO_3_H + 2H^+^ + O_2_ → 2 Cu(I)((S(CH_2_)_3_SO_3_H)_2_ +H_2_O_2_ (4)

Reaction (4) suggests that Cu(I) is bonded to the S-thiolate group.

The occurrence of a sodium adduct of SPS (SPS–Na) is natural because a commercially available SPS is manufactured in that form. On the other hand, complex Cu^2+^(SPS) was also crystallized and identified in solid state by Pasquale [22] as a product of reaction MPS with Cu^2+^ (Reaction 3). Pasquale reported that Cu^2+^ ions are formed during air oxidation and are bound to the sulfonate group. Our results obtained for the MPS solution (Samples 1, 3, 5, 7) show that the amounts of SPS–Na are proportional to the immersion times of both nitinols as well as copper substrates. The lower amount of SPS–Na for copper substrate may suggests that additional amounts of Cu^+^ allow formation of SPS–Cu that has been confirmed by distribution of SPS–Cu for Samples 5 and 7. In reality, fragment 371 assigned as Cu(II)-SPS can also correspond to Cu(I)[MPS]_2_ complex [22] as a product of reaction:

Cu(I)SC_3_H_6_SO_3_^−^+ SHC_3_H_6_SO_3_^−^ → Cu(I)[SC_3_H_6_SO_3_]_2_^−^ + H^+^ (5)

For Samples 9–16 we should expect a reaction between SPS and Cu^2+^ in the solution phase as it was reported (UV-VIS, NMR) [21,24,25].

For Samples 9, 11, 13, and 15 we identified significant amounts of SHC_3_H_6_SO_3_^−^ fragments (Figure 1b) assigned to the MPS molecule. For Samples 13 and 15 on the copper surface, MPS is yielded from Cu(I)MPS (a product of Reaction (3)). For Samples 9 and 11, prepared on the nitinol substrate, the fragment SHC_3_H_6_SO_3_^−^ is yielded by homolytical cleavage of S-S disulphide bonds in the SPS molecules.

Moreover, for Samples 9, 11, 13, and 15, the CuSC_3_H_6_SO_3_^−^ (*m/z* = 217) fragment was also identified and can be assigned to the complex Cu(I)SC_3_H_6_SO_3_^−^, the product of the reaction of SC_3_H_6_SO_3_^−^- (released from SPS during sputtering) with Cu^2+^ ions.

After rinsing (Samples 12, 14, 16, 18), there is a lack of Cu(I)SC_3_H_6_SO_3_ that is soluble in the water.

Moreover, the SPS molecules (Samples 16 and 18) after adsorption on copper immediately dissociate releasing two MPS molecules that immediately adsorb on the copper, remaining after rinsing as it was identified in the form CuS^−^. A similar behavioural pathway occurs for the unrinsed MPS/Cl and SPS/Cl samples.

Overall, for solutions consisting of SPS, two possible pathways of formation and decomposition of fragment 371 should be considered. The first pathway, known from the literature [12,44,45,46], relies on two the following stages: (1) disulphide bond in SPS molecule is cleaved into two MPS units, and (2) a reaction between MPS molecules and Cu^2+^ occurs according to Reaction (3). In the excess of MPS, a Cu(I)[SC_3_H_6_SO_3_]_2_^−^ complex (Reaction (5)) is formed. In the second pathway, the Cu^2+^(SPS) complex (identified previously by Pasquale [22]) in the layer is simply cleaved into CuSC_3_H_6_ SO_3_ and MPS. Considering that the last proposed scheme is only a single step we can claim that the existence of the Cu^2+^(SPS) complex is more favourable than Cu(I)[SC_3_H_6_SO_3_]_2_^−^.

Very low amounts of the SPS–Na (*m/z* = 331) were detected for unrinsed layers (Samples 21, 23, 29, 31) deposited on the copper surface from solutions containing Cl ions. In turn, the SPS–H fragment (*m/z* = 309) was not identified for Samples 21, 23, 29, 31. It suggests that the Cu–SPS complex is preferentially created over the sodium and protonated form of SPS in the chloride-consisting environment in near the copper surface in the crystallized phase. The affinity of Na^+^, H^+^, and Cu^2+^ ions for the sulfonate groups of SPS molecules can be estimated from the following reactions:

SPSNa + H^+^ → SPSH + Na^+^ (6)

SPSNa + Cu^2+^ → SPSCu + Na^+^ (7)

**Figure 2:**
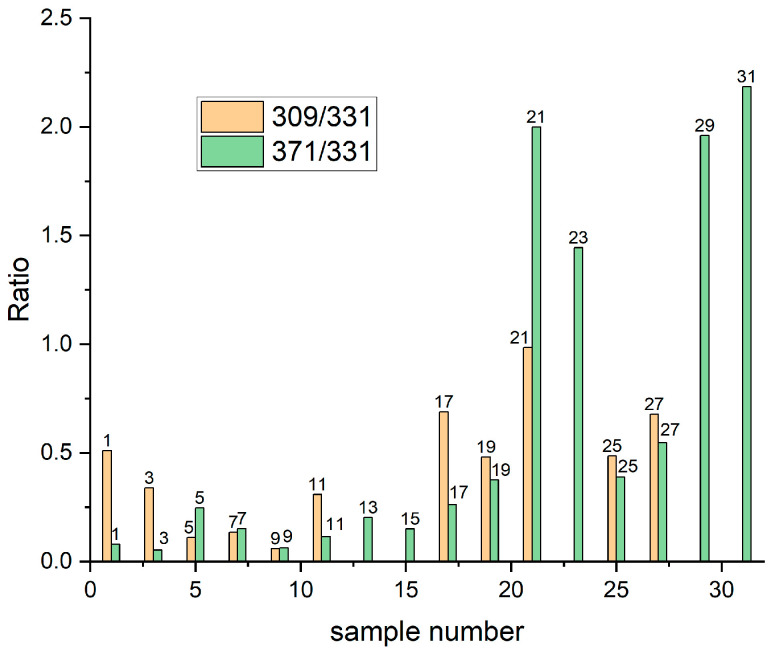
Distribution of the ratio intensity of the fragment 309/331 and 371/331 for 32 samples prepared in dip-coating experiment.

To estimate the shift of Reactions (6) and (7) the intensity ratio of fragments 309(SPSH)/331(SPSNa) and 371(SPSCu)/331(SPSNa) can be used. Assuming that C_SPSNa_ = 0.1 M = [H^+^] from sulphuric acid, we suppose that the shift of Reactions (6) and (7) to the right side occurred when the ratios 309/331 and 371/331 were higher than one, and to the left side for ratios lower than one. In Figure 2 the distributions of both 309/331 and 317/331 ratios for all unrinsed samples are shown. For Samples 25 and 27 we observed a significant shift in Reactions (6) and (7) to the right side after addition of chloride ions in comparison to Samples 9 and 11 containing only SPS. It is possible that chloride ions can form a pair Cl—Na^+^ that prevents the shift of Reactions (5) and (6) to the left side during crystallization. For Sample 25, based on Reactions (6) and (7), one mole of SPS–Na^+^ should react with 0.025 moles of Cu^2+^ and 0.025 moles of H^+^, providing 0.025 moles of SPS–H and 0.025 moles of Cu–SPS. Basically, two sulfonate groups of SPS can be protonated and cationizated by Cu^2+^, but as TOF-SIMS spectrometry only yielded single charged fragments, we only consider reactions with one sulfonate group.

In Samples 1 and 3 containing Cu(I)C_3_H_6_SO_3_ and SPS, all copper ions are complexed in the form MPS-Cu. The fragment Cu–SPS has the same mass as Cu[MPS]_2_, so we conclude that the 371 fragment forms in Reaction (5) as suggested previously by Cardona [24].

It this way the ratio 371/331 cannot be directly assigned to Reaction (5). Alternatively, the 371/217 ratio can be helpful in estimating the kinetics of Reaction (3).

The ratio of 371/217 has low values (ranges from 0.032 for Sample 3 to 0.067 for Sample 19). Chloride ions shift the steady state of Reaction (5) for samples immersed longer (ratio equal to 0.067 for Sample 19 and 0.032 for Sample 3), whilst for those with short immersion time changes are less significant (Sample 1—0.06 vs. Sample 17—0.05). On the other hand, for Sample 19 we observed very strong signals for SPS–H, SP-Na, and Cu[MPS]_2_, due to the long immersion time (60 s).

While analysing the layers deposited on the copper substrates (samples 21, 23, 29, and 31) we must consider that the amount of SPS in the solutions is significantly reduced due to the dissociative cleavage of disulphide bonds on the copper surfaces. Due to this reason, the intensity of SPSNa, SPS–H, and SPS is reduced, and it has an influence on the shift of Reactions (5) and (6). It can be clearly seen that Reaction (5) is shifted to the right side for Samples 21, 23, 29, and 31 containing Cl ions. For the same solutions but without chloride ions (Samples 5, 7, 13, and 15) the steady state is shifted to the left side. This unambiguously means that Cl ions are involved in the formation of Cu–SPS complexes in the layer deposited on the copper in the crystalline phase. On the other hand, we did not find fragments in the TOF-SIMS spectra that can be assigned to the Cu–SPS–Cl complexes.

The smaller mass fragments: CH_2_SO_3_^−^, CH_2_SO_3_H^−^, C_2_H_3_SO_3_^−^, and C_3_H_5_SO_3_^−^ were identified in all samples but they are less informative than high mass fragments. The intensity of C_2_H_3_SO_3_^−^ and C_3_H_5_SO_3_^−^ demonstrates a very similar distribution, whereas the distribution of the protonated form CH_2_SO_3_H is slightly different. The CH_2_SO_3_H^−^ fragment demonstrates a very good correlation with the CuS^−^ fragment. It can strongly suggest that for proximity of sulfonate group and copper substrate hydrogen radical transfer to the sulfonate end is efficient and gives high yield of CH_2_SO_3_H^−^. Moreover, the CH_2_SO_3_^−^ fragment can be yielded not only from Cu(0)SC_3_H_6_SO_3_H^−^ but also from fragments: Cu(I)MPS, Cu(II)SPS. Similarly, C_2_H_3_SO_3_^−^ and C_3_H_5_SO_3_^−^ fragments demonstrate similar distribution, slightly different than CH_2_SO_3_^−^, which may suggest that they are yielded from different geometrical conformation than CH_2_SO_3_^−^.

Sulphate ions were identified in the form: SO_4_^−^ and SO_4_H^−^. For the copper substrate for samples containing chloride ions (MPS/Cl and SPS/Cl) as well as SPS, the corresponding distribution of SO_4_^−^ and SO_4_H^−^ is similar showing minimum intensity for Samples 13–16, 21–24, and 29–32, meaning that after adsorption of MPS, SPS, and chloride ions there are no available adsorption sites for sulphate ions. On the other hand, for sample 31 sulphate ions can coexist with chloride and thiolate ions. This means that during dissociative adsorption of SPS competitive co-adsorption of sulphate and chloride ions occurs.

What is interesting is that for Samples 5 and 7 (MPS without chloride ions), adsorption of sulphate ions in the form SO_4_H^−^ takes place. On the contrary for SPS, (Samples 13 and 15) sulphate ions are not adsorbed. It can strongly support the mobility of the SPS species on the copper surface that was identified before dissociation onto two MPS units’ [10,45,46,47] block adsorption sites for sulphate ions. On the other hand, MPS are immediately bonded to the copper leaving a number of adsorption sites for sulphate ions.

The last very important fragment is CuSC_3_H_6_SO_3_^−^ (*m/z* = 217). This fragment can be assigned to: (1) Cu(0)SC_3_H_6_SO_3_, (2) Cu(I)C_3_H_6_SO_3_, and (3) -SC_3_H_6_SO_3_Cu(II). Fragment (1) is yielded from MPS molecules chemically adsorbed on the copper. Fragment (2) is the product of Reaction (3). The third fragment can be yielded from the SPS–Cu complex (*m/z* = 371) as well as from fragmentation of Cu[MPS]_2_^−^ (the product of Reaction (5)). In the latter case, it can be assigned to -Cu(I)C_3_H_6_SO_3_. Moreover, the CuSC_3_H_6_SO_3_^−^ fragment can interfere with persulfate ion NaS_2_O_8_H_2_^−^ with the same *m/z* = 217. Possible formation of persulphate ion may occur through the reaction of sulphate radical with hydrosulphate ion:

SO_4_^•−^+ HSO_4_^−^ → S_2_O_8_^2−^+ H^+^ + e^−^ (8)

and then thiolate radical reacts with hydrosulphate ions:

^•^SC_3_H_6_SO_3_^−^ + 2HSO_4_^−^ → S_2_O_8_^2−^ + 2SHC_3_H_6_SO_3_^−^ (9)

Thiosulfate ions S_2_O_8_^2−^ in the TOF-SIMS spectra are identified in the form NaS_2_O_8_H_2_^−^.

To evaluate the possibilities of this reaction occurring, in Reactions (8) and (9) we deposited layers on the nitinol surface from solutions: (1) 0.1 M Na_2_SO_4_; (2) 0.1 M Na_2_SO_4_ + 0.1 M H_2_SO_4_; (3) Na_2_SO_4_ + 0.1 M MPS; (4) Na_2_SO_4_ + H_2_SO_4_ + MPS; (5) Na_2_SO_4_ + SPS; (6) Na_2_SO_4_+H_2_SO_4_+SPS; (7) 0.1 M Na_2_S_2_O_8_; (8) MPS + H_2_SO_4_; (9) SPS + H_2_SO_4_; (10) 0.1 M SPS; and (11) 0.1 M MPS, by using the dip-coating method. In the TOF-SIMS spectra we detected a peak at *m/z* = 217 for Samples 2, 4, 6, 7, 8, 9. 

Identification of the peak at *m/z* = 217 for Sample 2 proves that during sputtering the Reaction (8) can occur. For the Samples 4, 6, 8, and 9 the existence of persulfate ion is determined by Reaction (9) as HSO_4_^−^ ions from dissociation of sulphuric acid and ^•^SC_3_H_6_SO_3_^−^ from MPS or SPS are available. Overall, the presence of sulphuric acid in the solution is necessary for Reactions (8) and (9) as a source of hydrosulphate ions.

Hopefully, the TOF-SIMS method allows for detection of isotopes that provide an additional tool for identification. The ratio intensity for CuSC_3_H_6_SO_3_^−^/^65^CuSC_3_H_6_SO_3_^−^ (*m/z* = 219) is equal to 60/32 whilst NaS_2_O_8_H_2_/Na^34^S_2_O_8_H_2_ (*m/z* = 219) is equal to 88/9. Based on isotopic analysis it is relatively easy to assign fragment *m/z* = 217 to CuSC_3_H_6_SO_3_ or NaS_2_O_8_H_2._

**Figure 3:**
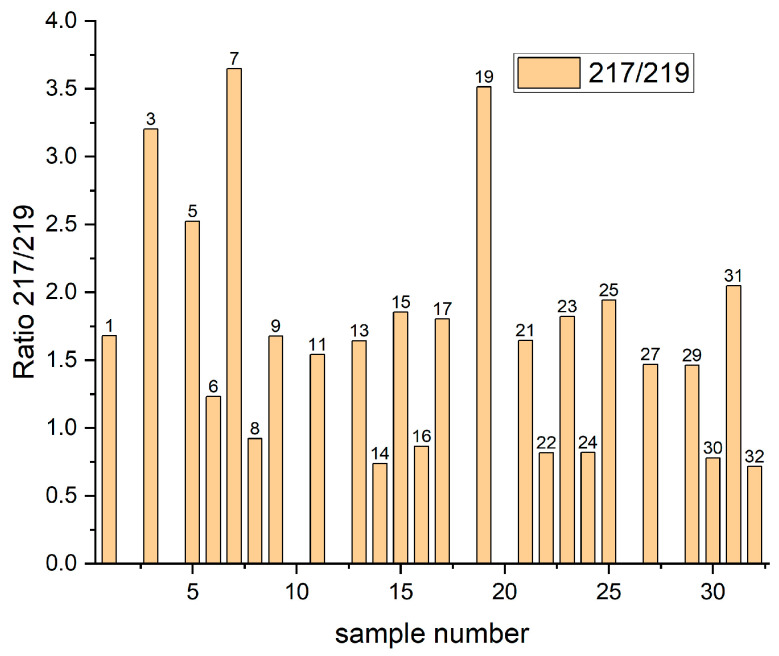
Distribution of the ratio intensity of the fragment 217/219 for 32 samples prepared in dip-coating experiment.

Figure 3 shows distribution of the intensity ratio of isotopes 217/219 for Samples 1–32. For Samples 2, 4, 10, 12 18, 20, 26, and 28 the *m/z* = 217 fragment was not detected. All these layers were deposited on the nitinol substrate and were rinsed, meaning that Cu(I)C_3_H_6_SO_3_ is highly soluble in water and is removed during rinsing.

The existence of *m/z* = 217 for rinsed samples on the copper substrate (6, 8, 14, 16, 22, 24, 30, 32) can be assigned to Cu(0)C_3_H_6_SO_3_ as MPS molecules that become chemically bonded to the copper and remain even during rinsing. We can observe (Figure 3) that the intensity of the 217/219 ratio for these samples is slightly below one, only for Sample 6 is the intensity ratio higher than one. It is not clear why the amount of fragment 219 is higher than 217 for mentioned samples. It seems that some unidentified fragments determine the growth of the intensity of fragment *m/z* = 219 for rinsed samples.

The unrinsed samples: 1, 9, 11, 13, 15, 17, 21, 23, 25, 27, 29, and 31 demonstrate similar, close to theoretical isotope distribution and we can assign the fragment with *m/z* = 217 to the ion Cu(I)SC_3_H_6_SO_3_^−^ as a product of Reaction (3). For Samples 1, 17, 21, and 23 no other reactions are possible. For Samples 9, 11, 25, and 27 the fragment *m/z* = 217 can be assigned to –SC_3_H_6_SO_3_Cu (II) that refers to Cu–SPS complex. Moreover, Samples 3, 5, 7, and 19 demonstrate a significantly higher ratio of 217/219, meaning that the CuSC_3_H_6_SO_3_^−^ fragment in this case is very likely overlapped by fragment Na_2_S_2_O_8_H_2_ corresponding to the thiosulfate ions S_2_O_8_^2^ produced in Reactions (8) and (9).

### 2.2. Cyclic Voltammetry Measurements—Copper Electrodeposition from Base Solution without and with Additives: Cl, MPS, and SPS

**Figure 4:**
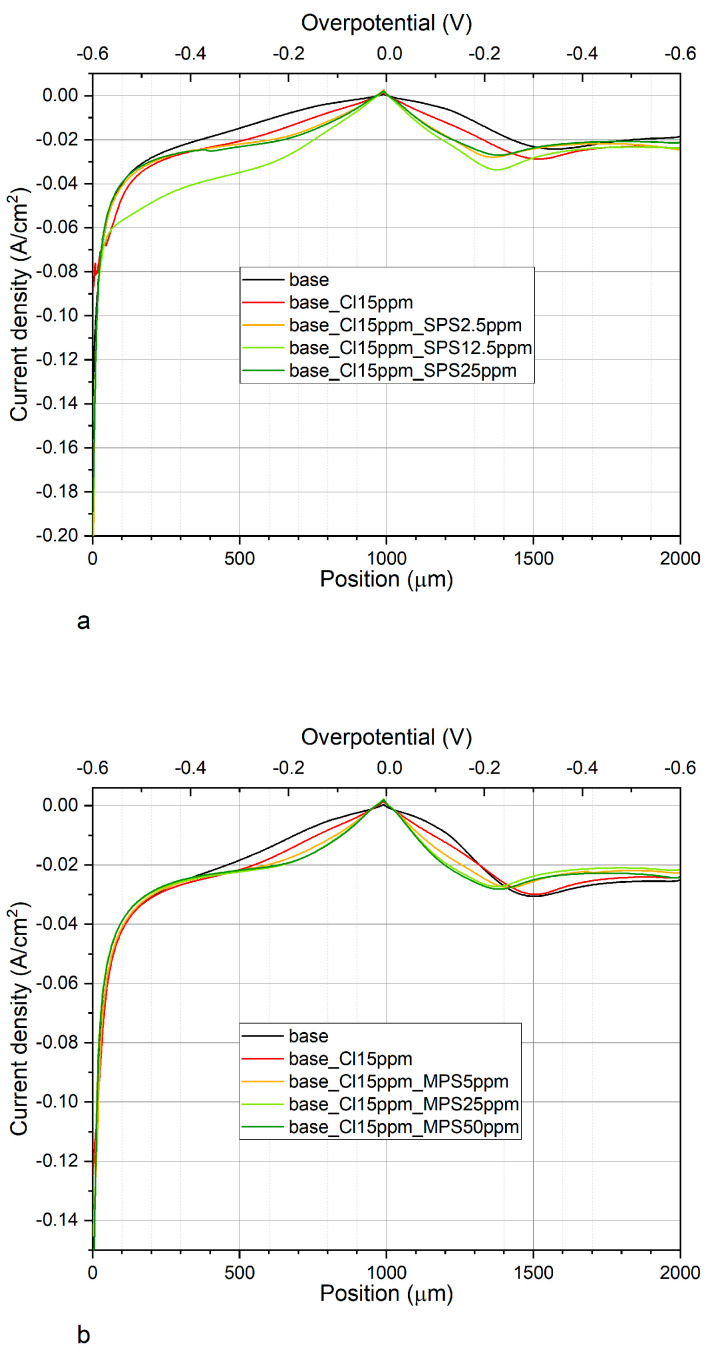
(**a**) Cyclic voltammetry curves recorded for base electrolyte, after addition of 15 ppm of Cl and SPS at the concentration 2.5, 12.5, and 25 ppm. (**b**) Cyclic voltammetry curves recorded for base electrolyte, after addition of 15 ppm of Cl and MPS at the concentration 5, 25, and 50 ppm. The wire position from 0 to 1 mm corresponds to the forward scan from −0.6 V to OCP (overpotential 0 V) while the wire position from 1 to 2 mm corresponds to the reverse scan from OCP to the −0.6 V.

**Figure 5:**
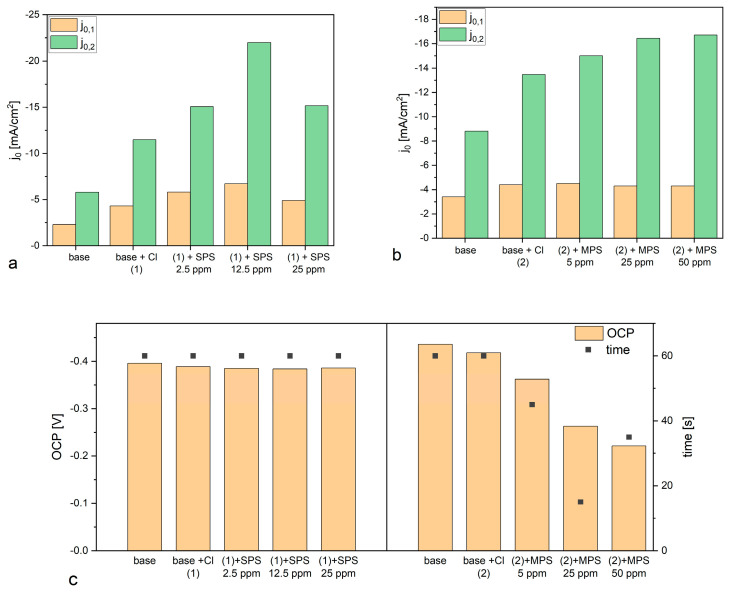
(**a**) Exchange current densities j_0,1_ and j_0,2_ for base, base + Cl, base + Cl + 2.5 ppm SPS, base + Cl + 12.5 ppm SPS, and base + Cl + 25 ppm SPS. (**b**) Exchange current densities j_0,1_ and j_0,2_ for base, base + Cl, base + Cl + 5 ppm MPS, base + Cl + 25 ppm MPS, and base + Cl + 50 ppm MPS. (**c**) Open circuit potential (OCP) and time required for stabilization of OCP for base, base + Cl, base + Cl + 2.5 ppm SPS, base + Cl + 12.5 ppm SPS, and base + Cl + 25 ppm SPS; and base, base + Cl, base + Cl + 5 ppm MPS, base + Cl + 25 ppm MPS, and base + Cl + 50 ppm MPS; respectively.

Figure 4a,b depicts CV curves while Figure 5a,b shows exchange current densities, obtained for base electrolyte, and after addition of Cl, SPS, or MPS at different concentrations. Figure 5a shows distribution of exchange current densities for the reduction of Cu^+^ to the metallic Cu (j_0,1_) and the reduction of Cu^2+^ to Cu^+^ (j_0,2_) for base solution and after addition Cl and SPS at different concentrations. The exchange current density j_0,1_ was calculated based on the CV curves by linear fitting of the CV curve for high overpotential region (η = −150 mV to −250 mV) while j_0,2_ by fitting the CV curve for very low overpotentials (η < −20 mV) for forward scans. It is widely assumed that the reduction of Cu^+^ to the metallic Cu is based on the inner-sphere mechanism [8,9].

On the other hand, the reduction of Cu^2+^ to Cu^+^ relies on the ion transport rather than electron transfer reactions and the ion migration reactions are determined by the reorganization of solvent and surface coverage [48]. As it is depicted in Figure 5a that addition of chloride ions as well as SPS in different concentrations to the base electrolyte increases the exchange current densities j_0,1_ and j_0,2_. However, the accelerating abilities are the greatest for Cl/SPS12.5ppm. Furthermore, the ratio of j_0,2_/j_0,1_ is varied in the following order: 2.51, 2.67, 2.6, 3.28, and 3.09 for base; and Cl, Cl/SPS2.5ppm, Cl/SPS12.5ppm, and Cl/SPS25ppm, respectively. This means that the accelerating abilities of Cl and Cl/SPS2.5ppm are determined by enhancement of inner-sphere reactions (j_0,1_) and ion migration reactions (j_0,2_) at the same level. Recently, it was suggested [8] that exchange current density j_0,2_ can strongly depend on working electrode rotation speed. Due to this reason, in the latter work [8] where the electrode rotational speed was fixed to 800 rpm, the ratio of j_0,2_/j_0,1_ was substantially different than in our work since we electrodeposited copper without stirring. For the highest concentrations of SPS (12.5 and 25.0 ppm) the ratio of j_0,2_/j_0,1_ significantly increases, this strongly suggests the ion migration reactions determined by solvent reorganization (dehydration sphere) and surface coverage dominate the inner-sphere reaction. Moreover, the greatest values of j_0,1_ and j_0,2_ were calculated for Cl/SPS12.5ppm.

Figure 5b depicts the distribution of exchange current densities j_0,1_ and j_0,2_ for base, and with the additions of Cl and Cl/MPS solutions. The j_0,2_/j_0,1_ ratio for the base electrolyte is similar to what was obtained in the previous experiment, when accelerating abilities of SPS were examined, while exchange current densities j_0,1_ and j_0,2_ demonstrate higher values. The reason for greater accelerating abilities is unknown. We examined the copper surface chemistry after electrodeposition by TOF-SIMS (see the next section) and the results prove that the solution did not consist of any impurities and traces of Cl/SPS from the previous experiment. Larger accelerating abilities might be determined by different surface roughness of nitinol substrate. After addition of Cl to the base electrolyte, the j_0,2_/j_0,1_ ratio value increases from 2.6 (obtained for base) to 3.06, and to 3.3 and 3.8 after addition of MPS5ppm, MPS25ppm, respectively. At the highest concentration of MPS (50 ppm) the j_0,2_/j_0,1_ ratio is similar as for MPS25ppm. In comparison to SPS, it can clearly be seen that after addition of MPS the exchange current density j_0,1_ is constant while j_0,2_ increases. It strongly suggests that in our experimental condition during copper electrodeposition (the electrolyte was not stirred) the accelerating abilities of MPS rely only on the ion migration reactions determined by the solvent reorganization. Distribution of open circuit potential (OCP) defined after deposition of the copper layer at constant current densities j = −20 mA/cm^2^ over 30 s is shown in Figure 5c. For electrolytes containing different concentrations of SPS, the OCP value and the evaluation time needed to reach steady state, OCP was constant. Interestingly, the OCP decreased as a function of MPS concentration while the evaluation time of OCP was minimal for MPS25 ppm.

Our work shows that under open circuit conditions, SPS weakly interact with copper surfaces. On the other hand, strong interaction/adsorption is observed for MPS molecules. The lowest OCP potential was observed for the electrolyte with the greatest MPS concentration (50 ppm) while steady state was reached the fastest for MPS25ppm. It seems that it determines the mechanism of electrodeposition that relies mainly on the ion migration reactions, while for the solution consisting of SPS, inner-sphere mechanisms also play significant role. The above discussion, which is based on the cyclic voltammetry curves, is widely used for investigations on the mechanism of copper electrodeposition in the presence of organic additives such as MPS and SPS [2,3,12,49]. On the other hand, Gileadi et al. [9] postulated that the Tafel slope extracted from the CV curve could not to be used to build a mechanistic model to distinguish different mechanisms of metal deposition.

Due to this reason, to gain more insight into the accelerating abilities of MPS and SPS we examined surface chemistry of samples by using the TOF-SIMS spectrometry and detailed discussion is provided in the next section.

### 2.3. Evaluation of the Molecular Composition inside the Electrodeposited Copper Layer

**Figure 6:**
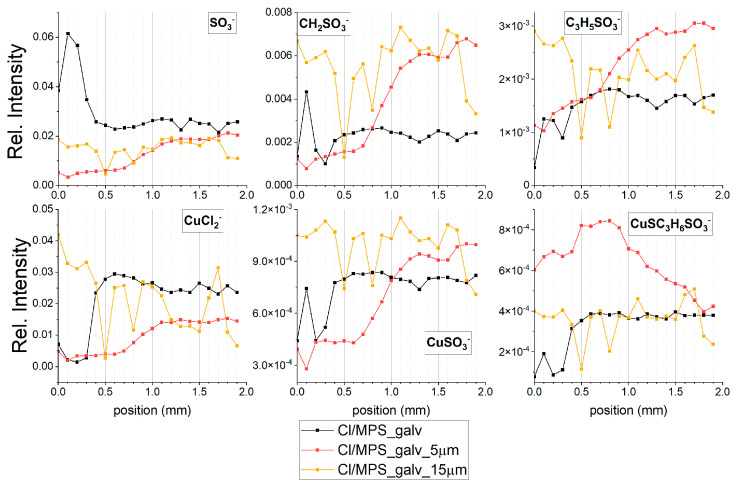
Distribution of intensity the fragments: SO_3_^−^, CH_2_SO_3_^−^, C_3_H_5_SO_3_^−^, CuCl_2_^−^, CuSO_3_^−^, and CuSC_3_H_6_SO_3_^−^ along the wire position identified on the copper surface electrodeposited from solution MPS/Cl assigned as Cl/MPS, after etching 5 µm of copper layer without ultrasonic (Cl/MPS_galv_5µm) and after etching 15 µm of copper with using ultrasonic (Cl/MPS_galv_15µm).

Figure 6 shows the distribution of CH_2_SO_3_^−^, C_2_H_3_SO_3_^−^, and CuSC_3_H_6_SO_3_^−^ along the wire before etching of copper, after etching 5 µm and 15 µm copper layers, respectively. The intensity of CH_2_SO_3_^−^, C_2_H_3_SO_3_^−^ and C_3_H_5_SO_3_^−^ fragments after etching without using ultrasounds is comparable to a wire length of 0–2 mm while in the region of 3–6 mm it was significantly greater than before etching. However, we must keep in mind that the TOF-SIMS data depicted in Figure 6 do not correspond exactly to the same areas of samples determined before etching. Nevertheless, the level of intensity of thiolate fragments is significantly high and it proves that thiolate molecules are incorporated into the copper deposit. After application of ultrasounds the intensity demonstrates more expressive fluctuation, while the intensity is roughly comparable to the layer after etching without the use of ultrasounds. Intensity of CuSC_3_H_6_SO_3_^−^ fragment is the highest in the region 0–3 mm and then gradually decreases after etching without ultrasound. It seems that a specific orientation of molecules exposes a higher number of thiolate molecules on the surface in the 0–3 mm region, which in turn yields larger abundance of CuSC_3_H_6_SO_3_^−^ and less CH_2_SO_3_^−^ and C_3_H_5_SO_3_^−^ fragments. In the wire region of 3–6 mm, the lower intensity of CuSC_3_H_6_SO_3_^−^ and higher of CH_2_SO_3_^−^ and C_3_H_5_SO_3_^−^ can suggest that a greater number of thiolate molecules were incorporated into the copper layer than exposed on the copper surface.

The most important conclusion from the etching experiment is that thiolate molecules were incorporated into the deposit during copper electrodeposition. Moreover, the intensity of chloride ions demonstrates similar distribution to the intensity of thiolate ions, meaning that both thiolate and chloride ions are incorporated into copper, but the amounts of thiolate and chloride ions are different. After etching, we observed a significant decrease in the intensity of chloride ions. This means that the relative Cl/thiolate ratio in the deposit is lower than on the surface, which means that thiolate molecule incorporation into the copper layer is higher than that of chloride ions.

Inclusion of thiolate into the Cu deposit was previously studied using XPS [27]. However, the procedure of deposition was different (in comparison to the procedure used by us): firstly, a gold electrode was immersed in 5 ppm of SPS solution and then transferred to a solution containing CuSO_4_, PEG, and chloride ions for copper electrodeposition. In this way, the re-adsorption and incorporation of thiolate into the copper deposit was studied. No studies on possible chloride incorporation into deposit were carried out. It was concluded that a transfer of small portions of thiolates from the interface Au/Cu to the top of the growing Cu layer occurred because thiolate re-adsorption is kinetically controlled. The authors claim that the amount of transferring thiolate is sufficient to accelerate Cu growth, and SPS is not required in the solution. This important and controversial conclusion was evaluated in the next section of this work.

### 2.4. Examination of SPS Re-Adsorption and Accelerating Abilities in the Case of Lack SPS in the Solution

**Figure 7:**
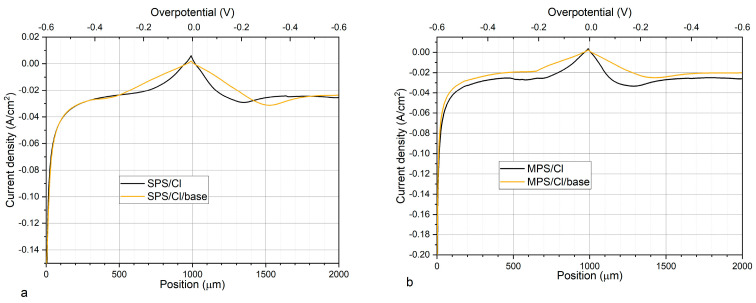
(**a**) Cyclic voltammetry curves recorded for copper electrodeposited from solution containing SPS (25 ppm) + Cl (15 ppm) and after transferring that sample into base electrolyte (SPS/Cl/base). (**b**) Cyclic voltammetry curves recorded for copper electrodeposited from solution containing MPS (50 ppm) + Cl (15 ppm) and after transferring that sample into base electrolyte (MPS/Cl/base).

Figure 7a,b depict CV curves that demonstrate accelerating abilities for samples SPS/Cl and MPS/Cl. Accordingly, the exchange current density values j_0,2_ were equal to 16.7 and 17.8 mA/cm^2^, respectively. After transferring the received copper to the base electrolyte exchange current, densities j_0,2_ were 5.6 and 5.8 mA/cm^2^, respectively, meaning that the accelerating abilities were lost.

**Figure 8:**
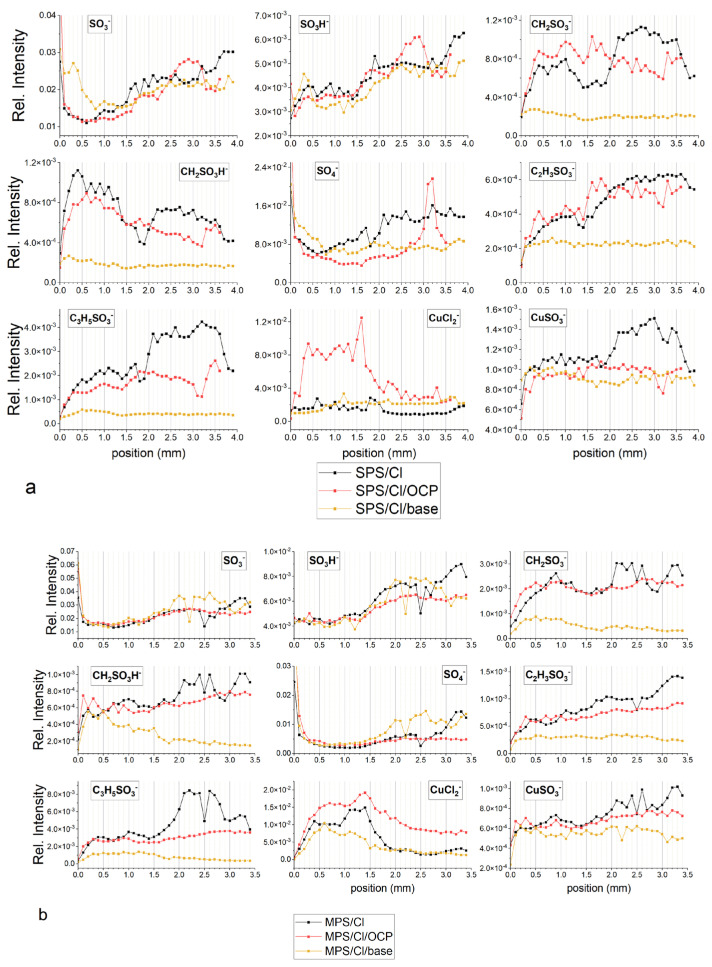
(**a**) Distribution of intensity of the fragments: SO_3_^−^, SO_3_H^−^, CH_2_SO_3_^−^, CH_2_SO_3_H^−^, SO_4_^−^, C_2_H_3_SO_3_^−^, C_3_H_5_SO_3_^−^, CuCl_2_^−^, and CuSO_3_^−^ along the wire position identified on the copper surface electrodeposited from solution containing SPS/Cl, after transferring that sample into base solution under open circuit potential condition SPS/Cl/OCP and after transfer for electrodeposition to the base solution SPS/Cl/base. (**b**) Distribution of intensity of the fragments: SO_3_^−^, SO_3_H^−^, CH_2_SO_3_^−^, CH_2_SO_3_H^−^, SO_4_^−^, C_2_H_3_SO_3_^−^, C_3_H_5_SO_3_^−^, CuCl_2_^−^, and CuSO_3_^−^ along the wire position identified on the copper surface electrodeposited from solution containing MPS/Cl, after transferring that sample into base solution under open circuit potential condition MPS/Cl/OCP and after transfer for electrodeposition to the base solution MPS/Cl/base.

The beforementioned results could mean that a monolayer of thiolate molecules on the copper surface received during electrodeposition from the electrolyte MPS/Cl does not play an accelerating role. In consequence, due to lack of MPS and Cl additives in the base, electrolyte accelerating abilities were completely suppressed. High accumulation of thiolate ions on the copper surface after electrodeposition from the MPS/Cl electrolyte is shown in Figure 8a. Accordingly, after immersing the sample to the base solution, thiolate coverage remains roughly unchanged, while chloride coverage slightly increases (MPS/Cl/OCP). This could mean that MPS units and chloride ions on the copper surface are fully re-adsorbable under OCP conditions. On the other hand, the amount of thiolate ions (CH_2_SO_3_^−^, C_2_H_3_SO_3_^−^, C_3_H_5_SO_3_^−^) was reduced by about three times and chloride ions by about two times after the subsequent layer’s deposition from the base electrolyte. Moreover, the amount of thiolate ions expressed as total thiols in a forward scan was maintained, which means that entire thiolate molecules were incorporated during copper electrodeposition. Then, these molecules were immediately buried and overbuilt by the copper layer electrodeposited from the base solution while the remaining monolayer of MPS/Cl that was not previously incorporated into copper demonstrated full re-adsorption properties. Similar behaviour was observed for SPS/Cl solution (Figure 8b).

**Figure 9:**
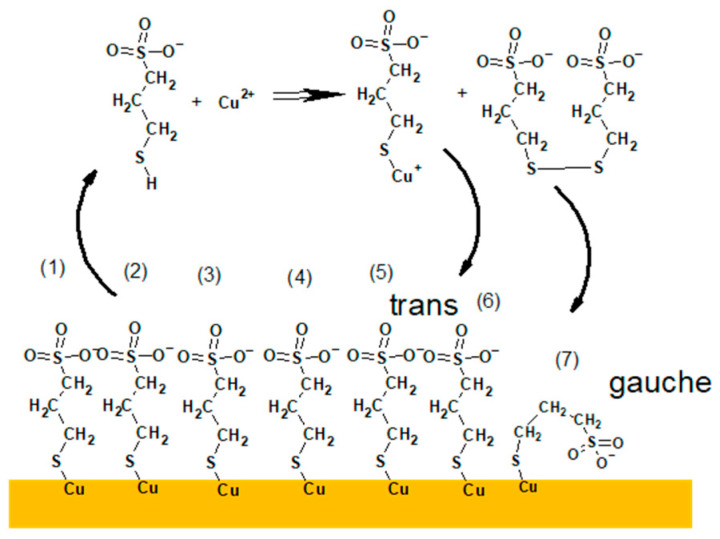
The proposed molecular arrangement of MPS monolayer on the copper surface during copper electrodeposition from the base solution on the previously pre-plated copper from MPS/Cl solution. Over the monolayer, reactions between desorbed MPS molecule and Cu^2+^ ions occur. The products of these reactions are re-adsorbed onto copper.

The proposed mechanism of adsorption/re-adsorption of thiolate molecules on the copper surface from the electrolyte not containing MPS and chloride ions (base electrolyte) is shown in Figure 9.

The MPS/Cl monolayer not incorporated into copper can be re-adsorbed as follows. After MPS units’ desorption and attaching of hydrogen ions (from the sulphuric acid) they react with Cu^2+^ ions, causing Cu(I)MPS complex and SPS (Reaction (3)) as shown in Figure 9. In the next step, Cu^+^ from the Cu(I)MPS complex can be electrochemically reduced to metallic copper while the released MPS molecules are adsorbing on the copper. Similarly, SPS molecules are able to dissociatively adsorb on the copper, providing two MPS units. The adsorption/re-adsorption occurs in the presence of chloride ions that also adsorb/re-adsorb on the copper surface. The partial desorption of chloride and desorption/re-adsorption of thiolate ions in the presence of SPS in the solution was examined and proven by STM studies [12]. The chloride adlayer defects are induced by copper electrodeposition and play a crucial role in MPS molecule re-adsorption. A significant drawback of latter STM experiments is that the solution consisted only of Cl, MPS, or SPS, but without copper sulphate. In our experimental conditions when the solutions with high content of copper sulphate were used, all MPS molecules are desorbed and re-adsorbed as shown in Figure 8a. This strongly suggests that without additives (MPS/SPS/Cl) in the solution, even under current flow, the dynamics of adsorption/re-adsorption are similar to those under OCP conditions. In this scenario, if MPS molecules only develop a monolayer, they are not able to incorporate into the copper deposit due to fast desorption/re-adsorption dynamics. Without incorporation of MPS into copper, there are very limited amounts of sulfonate groups in close proximity to the copper surface. Consequently, it diminishes the number of effective Cu^2+^–SO_3_^−^ ion pair interactions. The recent interaction is considered a crucial tool in accelerating abilities of MPS by dehydration of Cu^2+^ [8]. Moreover, the existence of Cu(I)MPS on copper surface may reduce the accelerating abilities of MPS molecules in the gauche orientation.

To examine the mechanism of adsorption/re-adsorption of MPS/SPS from a solution consisting of MPS/SPS/Cl, we conducted the appropriate experiment (described in detail in the next section).

### 2.5. TOF-SIMS Measurements—Copper Electrodeposition from Base Solution with and without Additives: Cl, MPS, and SPS

**Figure 10:**
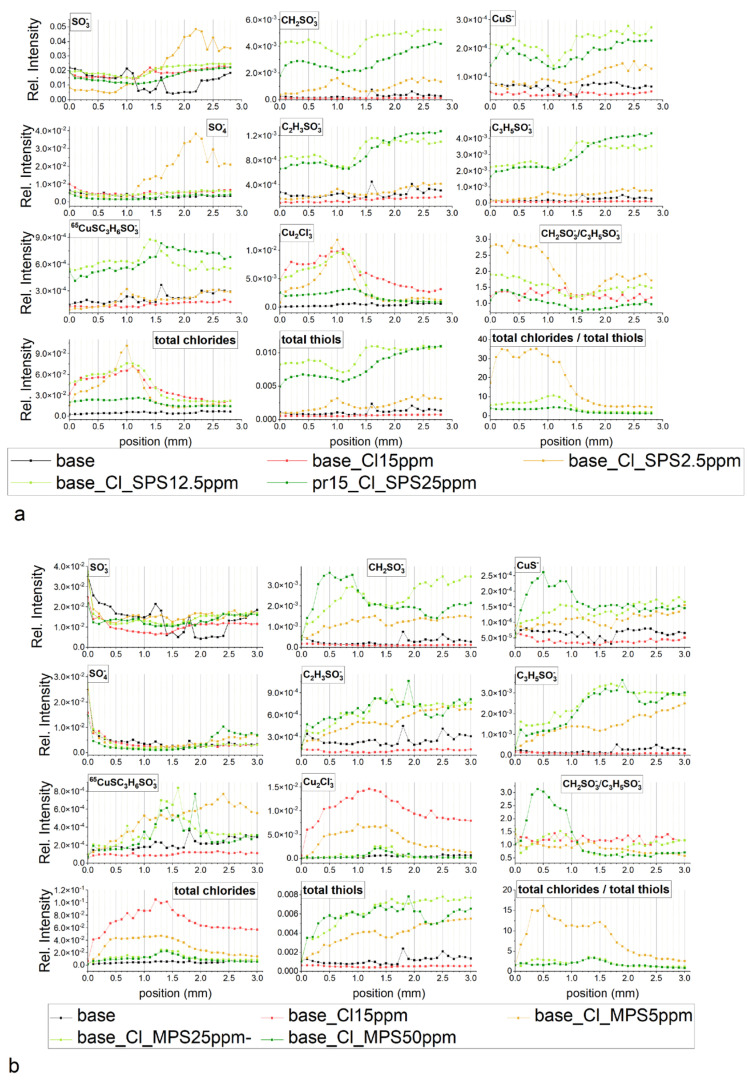
(**a**) Distribution of intensity of the fragments: SO_3_^−^, CH_2_SO_3_^−^, CuS^−^, SO_4_^−^, C_2_H_3_SO_3_^−^, C_3_H_5_SO_3_^−^, CuSC_3_H_6_SO_3_^−^, Cu_2_Cl_3_^−^, the ratio CH_2_SO_3_^−^/C_3_H_5_SO_3_^−^, total chlorides total thiols and the ratio total chlorides/total thiols along the wire position for (**a**) base electrolyte, base + 15 ppm Cl, base + 15 ppm Cl + 2.5 ppm SPS, base + Cl + 12.5 ppm SPS and base + Cl + 25 ppm SPS. (**b**) Distribution of intensity of the fragments: SO_3_^−^, CH_2_SO_3_^−^, CuS^−^, SO_4_^−^, C_2_H_3_SO_3_^−^, C_3_H_5_SO_3_^−^, CuSC_3_H_6_SO_3_^−^, Cu_2_Cl_3_^−^, the ratio CH_2_SO_3_^−^/C_3_H_5_SO_3_^−^, total chlorides total thiols and the ratio total chlorides/total thiols along the wire position for (**b**) base electrolyte, base + 15 ppm Cl, base + 15 ppm Cl + 5 ppm MPS, base + Cl + 12.5 ppm MPS and base + Cl + 25 ppm MPS. The wire position corresponds to the applied overpotential during CV experiment.

Figure 10a depicts the TOF-SIMS data of selected negative ions distributed along the wire length for samples obtained from the base electrolyte (black line), and solutions consisting of 15 ppm Cl (red) and 15 ppm Cl, with the addition of 2.5 ppm SPS (yellow), 12.5 ppm SPS (light green), and 25 ppm SPS (dark green). Relevant CV curves were shown and analysed in Section 2.2. The forward scan in the anodic direction (more positive potential, wire position 0–1 mm) corresponds to the adsorption regime, as we can expect an increase of adsorption of additives during a shift potential to OCP. On the other hand, the reverse scan in the cathodic direction (wire position 1–2 mm) corresponds to the desorption part, as we can expect that desorption should be accelerated, when potential becomes more negative. The chloride ions were identified as CuCl^−^, CuCl_2_^−^ and Cu_2_Cl_3_^−^ fragments while thiolate ions were identified as CuS^−^, CH_2_SO_3_^−^, C_2_H_3_SO_3_^−^, C_3_H_5_SO_3_^−^, and CuSC_3_H_6_SO_3_^−^.

In the dip-coating experiment (Section 2.1), it was demonstrated that the Cu(I)SC_3_H_6_SO_3_^−^ complex was identified as a fragment CuSC_3_H_6_SO_3_^−^ and was deposited as a thin layer on the copper surface; it is highly soluble after rinsing in an optimized way (see details in experimental section). About 20–30% of the primary amount of Cu(I)SC_3_H_6_SO_3_ complex still remained on the copper surface and could be identified (Figure 1a). The CuS^−^ fragment corresponds to the Cu–S bond and is yielded from adsorbed thiolate molecules on the copper surface. Potentially, the CuS^−^ fragment may also be yielded from the Cu(I)C_3_H_6_SO_3_ complex. However, the previous dip-coating experiment demonstrates that the CuS^−^ fragment is mainly yielded from the thiolate molecules that are chemically bonded to the copper surface. This is determined by the fact that after rinsing of Samples 5 and 7 (Cu(I)C_3_H_6_SO_3_ on the copper surface) in the dip-coating experiment, CuS^−^ intensity significantly increases while CuSC_3_H_6_SO_3_^−^ intensity decreases (Samples 6 and 8; Figure 1a). A similar observation was made for MPS/Cl solutions by comparing Samples 21/22 (rinsed) and 23/24 (rinsed), respectively.

The existence of the CuS^−^ fragments proves that MPS molecules are bonded to the copper by the thiolate end, which is fully consistent with previous results [35,45,46], as well as our dip-coating experiment (Section 2.1).

The increase in potential towards the anodic direction for the forward scan was accompanied by an increase in the surface coverage of chloride ions, since the intensity of CuCl_2_^−^ and Cu_2_Cl_3_^−^ increased (red line, Figure 10a). After injection of 2.5 ppm SPS (yellow line, Figure 10a), chloride surface coverage was reduced for nearly the whole potential and demonstrated stronger deviations in a function of applied potential. For the potential region corresponding to the 0–1.2 mm position, intensity of chloride significantly increased, while the rest of the potential region of the reverse scan exponentially reduced in similar way. In the dip-coating experiments (Section 2.1) it was suggested that CuCl_2_^−^ ions can be a good marker of chloride ions, as well as of metallic copper. However, it is important question how to distinguish between a chloride adlayer and uncovered copper metallic surface for samples received in CV experiments. Figures S2–S4 (Supplement Materials) show that increases in the intensity of the thiolate (CH_2_SO_3_^−^, C_3_H_5_SO_3_^−^) and chloride (CuCl_2_^−^) fragments are accompanied by a decrease in the intensity of Cu^−^. This is consistent with the dip-coating experiment (see Figure 1) and unambiguously proves that with the presence of a thiolate and chloride monolayer the area of the uncovered copper surface is reduced. In this way, the CuCl_2_^−^ fragment can be unambiguously assigned to the Cl^−^ adlayer.

Thiolate coverage is identified by the fragments: CuS^−^, CH_2_SO_3_^−^, C_2_H_3_SO_3_^−^, C_3_H_5_SO_3_^−^, and CuSC_3_H_6_SO_3_^−^. For high overpotentials (0–0.3 mm) the total thiolate surface coverage (Figure 10a) was rather low and stable, while significant increases and synergy effect with chloride ions was observed for lower overpotentials (region 0.4–1.0 mm) that exhibit significant increases in thiolate coverage and chloride ions.

The turning point was observed after switching to the reverse scan (position 1.1 mm), when strong desorption of chloride and thiolate ions occurred while replacement of the desorbed chloride ions by sulphate ions was observed at position 1.2 mm (η = −120 mV). For the rest of the range of the reverse scan of chloride ions, desorption continued while desorption of thiolate ions was stopped at wire position 1.4 mm (η = −240 mV), accompanied by a slight increase (at position 1.5–2.0mm).

To compare the adsorption coverage of chloride to that of thiolate ions, the intensity of all forms of chloride (CuCl^−^, CuCl_2_^−^, and Cu_2_Cl_3_^−^) and thiolate fragments (CH_2_SO_3_^−^, CuS^−^, C_2_H_3_SO_3_^−^, C_3_H_5_SO_3_^−^, and CuSC_3_H_6_SO_3_^−^) were added up separately and assigned as total chlorides and total thiols, respectively.

A high and rather stable ratio of Cl/thiols for the wire position 0.2–0.8 mm (Figure 10a) corresponds quite well to greater accelerating properties in comparison to the solution consisting of only chloride ions (see CV curve, Figure 4a). Subsequently, the ratio Cl^−^/thiols decreases and reaches a lower value than was observed for the corresponding overpotential for the forward scan in the 0.9–1.4 mm range. For lower overpotentials (0.8–1.2 mm), when strong co-adsorption of Cl and thiolate occurs, adsorption of thiolate is slightly dominated over chloride adsorption. For the reverse scan, when co-desorption started (1.1–1.2 mm) in a narrow potential window (−60 to −120 mV), the desorption rates of Cl^−^ and thiolate were similar, while for the rest potential region (1.3–2.0 mm), chloride ions were significantly more strongly desorbed than thiolate. It is interesting that the CH_2_SO_3_^−^/C_3_H_5_SO_3_^−^ ratio demonstrates roughly similar behaviour to the ratio of Cl/thiols. For the forward scan, the amount of CH_2_SO_3_^−^ fragments dominated over C_3_H_5_SO_3_^−^ and exhibited a rather stable CH_2_SO_3_^−^/C_3_H_5_SO_3_^−^ ratio value around 2.7 while for the reverse scan the CH_2_SO_3_^−^/C_3_H_5_SO_3_^−^ ratio was significantly reduced. Recently, SHINER studies [8] showed that the conformation gauche/trans ratio for the SPS/Cl system is strongly dependent on the applied potential. We also tried to evaluate the ratio of gauche/trans by Raman spectroscopy, and while the received Raman spectra were not reproducible and consisted of very regular background peaks that overlapped with the characteristic peaks, they did correspond to the thiolate gauche and trans conformations (results not shown). Due to this reason, full reproducibility of the whole potential range using Raman spectroscopy for our samples was not possible.

On the other hand, to the best of our knowledge, TOF-SIMS spectrometry has not been used for the determination of conformation changes in the adsorbed thiolate monolayer. At this point, we can safely assume that the CH_2_SO_3_^−^/C_3_H_5_SO_3_^−^ ratio can be used as an indicator of the ratio of gauche/trans conformation. However, the CH_2_SO_3_^−^/C_3_H_5_SO_3_^−^ ratio is not only determined by the total thiolate surface coverage depicted in Figure 10a, since we should expect that the greater the total surface coverage the lower the gauche/trans ratio. However, under low thiolate coverage as took place for 2.5 ppm SPS, adsorbed thiolate molecules can rather be isolated by greater chloride ions and the gauche conformation can be easily attained in these molecular arrangements. Previously, it was shown that chloride coverage and distribution of pitting in the chloride adlayer can be determined by selective desorption of chloride ions in a function of applied potential, which plays an important role in reorientation of thiolate molecules [12]. This fact can explain the significant reduction in CH_2_SO_3_^−^/C_3_H_5_SO_3_^−^ ratio for the reverse scan in the cathodic direction. During strong co-adsorption of chloride and thiolate ions (position 0.8–1.0 mm) due to the ordering of thiolate molecules, the contribution of trans conformation increases as the intensity of the C_3_H_5_SO_3_^−^ fragment rises more than that of CH_2_SO_3_^−^. Moreover, during co-desorption (1.1–1.4 mm) thiolates in gauche conformation are preferentially desorbed over trans conformation. What it is also important is that total chloride ions are also strongly desorbed during the reverse scan. This result determined the decreases in CH_2_SO_3_^−^/C_3_H_5_SO_3_^−^ratio, since Cl ions are replaced by thiolate ions.

In the TOF-SIMS spectra, we did not find fragments that corresponded to the Cu–MPS–Cl complexes in any form of copper (metallic, Cu^+^, or Cu^2+^) on the electrode surface. Moreover, in the solution the only evidence was the presence of MPS–Cu^+^–Cl complexes, which was supported by spectroscopic data [3], while other investigations [24] were in contradiction to this finding, demonstrating only the presence of Cu(I)MPS complexes. The latter finding was also supported by other investigations [4]. On one hand, we cannot definitely rule out the occurrence of Cu^+^(Cu^2+^)/MPS/Cl^−^ complexes. Furthermore, electrostatic interaction of sulfonate ends with Cu^2+^ cannot be directly examined by TOF-SIMS spectrometry due to lack of strong chemical Cu^2+^/SO_3_^−^ bonds. What is important, is that we also did not find any evidence of existing Cu–MPS–Cl complexes in our dip-coating experiment (Section 2.1). The latter observation provides more arguments against the existence of Cu–MPS–Cl complexes in the solution.

In order to gain more insight into the chemical state on the copper electrode, we performed CV experiments with greater SPS concentrations (12.5 ppm and 25 ppm) in the electrolyte solution.

After an injection of 10 ppm of SPS (total concentration of SPS 12.5 ppm) we observed a strong enhancement in accelerating abilities for the whole potential range (see discussion in the previous section) and significantly greater thiol coverage (light green curve) for the forward as well as reverse scan. For position 0.0–0.7 mm, total thiolate coverage is rather stable, then gradually decreases reaching minimum around 1.1 mm (overpotential η = −60 mV). Consecutively, thiolate coverage increases up to 1.5 mm and then is stable in the rest of potential range (position 1.5–2.0 mm). After switching off the current, under OCP conditions (position 2.0–2.8 mm) total thiol coverage is similar as observed at position 2.0 mm. Distribution of intensity of chloride ions in the range 0.7–2.0 mm is negatively correlated with thiolate, the greater chloride the lower thiolate coverage. It means that thiolate ions are partially replaced by chloride ions for low overpotential range during the forward scan while during the reverse scan in the region 1.2–2.0 mm, desorption of chloride ions is accompanied by thiolate adsorption. In comparison to solution consisting of 2.5 ppm SPS, chloride coverage is greater for the forward scan only while around OCP, while at position 0.9–1.0 mm is slightly lower. Moreover, the maximum chloride coverage at SPS 12.5 ppm is shifted to −60 mV for the reverse scan in comparison to solution of SPS 2.5 ppm. On the other hand, chloride coverage is very similar to the solution consisting of only chloride ions. Sulphate coverage is maintained on the low value for the forward as well as reverse scan, demonstrating an inverted distribution to chloride ions. The greater chloride coverage the lower sulphate ion coverage and vice versa.

After adding the next amount of SPS (total SPS concentration 25 ppm), thiolate and chloride coverage as well as the ratio chloride/thiolate is significantly lower than observed for 12.5 ppm SPS. This strongly suggests that the optimal molar ratio SPS/Cl is required for greater adsorption of Cl and SPS. For 25 ppm of SPS concentration, chloride and thiolate surface coverage is reduced while thiolate molecules demonstrate greater adsorption than chloride ions. The lower accelerating abilities may also relate to higher amounts of Cu(I)SC_3_H_6_SO_3_ accumulated over the thiolate layer. In this scenario, Cu(I)SC_3_H_6_SO_3_ plays the suppression role by blocking the access of Cu^2+^.

For 12.5 and 25 ppm SPS, the intensity of CuSC_3_H_6_SO_3_^−^ fragments demonstrate a stable value around OCP (the region 0.7–1.2 mm). The intensity of fragments CH_2_SO_3_^−^, C_2_H_3_SO_3_^−^, C_3_H_5_SO_3_^−^, and CuS^−^ in that potential region decreases. This is especially visible at 12.5 ppm SPS for CH_2_SO_3_ and CuS^−^. Different distribution of CuSC_3_H_6_SO_3_^−^ and CH_2_SO_3_^−^, C_2_H_3_SO_3_^−^, C_3_H_5_SO_3_^−^, and CuS^−^ suggests that the fragment CuSC_3_H_6_SO_3_^−^ corresponds to the Cu(I) thiolate complex while the fragments CH_2_SO_3_^−^, C_2_H_3_SO_3_^−^, C_3_H_5_SO_3_^−^, and CuS^−^ to the adsorbed thiolate molecules. It means that in this potential region, a significant amount of desorbed MPS molecules is converted into the Cu(I)SC_3_H_6_SO_3_ complex while the amount of the SPS is significantly reduced and this reaction is accelerated by the chloride ions.

Even more so, for SPS 12.5 ppm and SPS 25 ppm the ratio CH_2_SO_3_^−^/C_3_H_5_SO_3_^−^ is significantly lower than was observed for SPS 2.5 ppm and exhibits a tendency to decrease during the reverse and forward scan. It is rational to assume that under greater thiolate and chloride surface coverage, the contribution of gauche conformation is significantly reduced, since adsorbed MPS molecules are tightly surrounded by chloride ions. On the other hand, the ratio of CH_2_SO_3_^−^/C_3_H_5_SO_3_^−^ is unexpectedly greater for higher thiolate and chloride coverage (SPS 12.5 ppm) than observed in lower thiolate and chloride coverage (SPS 25 ppm). It strongly suggests that chloride ions play an important and positive role in the gauche orientation of thiolate molecules, even under high thiolate and chloride coverage.

This observation is supported by the chloride/thiol intensity ratio that demonstrates the same behaviour as the CH_2_SO_3_^−^/C_3_H_5_SO_3_^−^ ratio, which exhibits a decreasing trend as SPS concentration increases.

Figure 10b depicts distribution of characteristic fragments for base solution, after addition of chloride ions and MPS at concentrations of 5, 25, and 50 ppm, respectively. Similarly, as observed in the experiment with the addition of SPS, the copper surface deposited from the base electrolyte does not contain chloride or thiolate ions. After addition of chloride ions, the fragment Cu_2_Cl_3_^−^ demonstrates greater intensity as observed in the experiment with the addition of SPS. The maximum intensity of chloride ions is observed around OCP.

After addition of 5 ppm MPS, the intensity of chloride ions is significantly reduced for the whole range of overpotentials for forward and reverse scans. Similarly, the intensity of total thiols demonstrates greater value for the whole potential range of forward and reverse scans. This is different behaviour than was observed for 2.5 ppm SPS, when the distribution of intensity of chloride was more positively correlated with thiolate ions. Furthermore, the ratio of total chloride/total thiols is about two times lower than was observed for 2.5 ppm SPS.

The ratio CH_2_SO_3_^−^/C_3_H_5_SO_3_^−^ is nearly constant in the 0.0–1.2 mm range, while greater overpotentials slightly increases during the reverse scan. The exchange current density j_0,2_ only slightly increases from 13.5 mA/cm^2^ (Cl) to 15 mA/cm^2^ for MPS 5 ppm. On the other hand, exchange current density j_0,1_ remains unchanged while for SPS 2.5 ppm, j_0,2_ rises from 11.5 mA/cm^2^ (Cl only) to 15 mA/cm^2^ for SPS 2.5 ppm. The most important difference between MPS and SPS at the lowest concentration is that chloride ions are strongly co-adsorbed with SPS ions in the range of low overpotentials (η < −120 mV). On the other hand, chloride ion coverage is stable in the broad potential range (0.3–1.2 mm) while thiolate coverage increases. Synergetic co-adsorption of chloride and SPS may be determined by preferential changes in the surface roughness (crystal orientation) of electrodeposited copper in the presence of Cl/SPS, which in turn increases the available adsorption centres for chloride ions. Moreover, the binding energy of SPS onto copper is lower than for MPS [12] that may allow the formation of Cu^+^–SPS^−^–Cl^−^ complexes on the near-electrode surface under electrodeposition conditions. Determination of open circuit potential (see the previous section) demonstrates and proves the great adsorption abilities of MPS and weak direct interactions of SPS with copper without an external current source.

At greater MPS concentrations (25 and 50 ppm), chloride surface coverage demonstrates very similar distribution and is significantly reduced in comparison to the MPS 5 ppm. Thiolate surface coverage of CH_2_SO_3_^−^, C_2_H_3_SO_3_^−^, and C_3_H_5_SO_3_^−^ CuS^−^ increases and demonstrates significantly different distribution while the intensity of CuS^−^ C_3_H_5_SO_3_^−^ is more positively correlated with the distribution of chloride. For MPS 25 ppm, the intensity of the fragment CH_2_SO_3_^−^ rapidly grows at the highest potentials (0.0–0.3 mm) then is stable and decreases from 0.8 mm. On the other hand, the intensity of CH_2_SO_3_^−^ is similar in the 0.8–1.3 mm range, while for position 0.0–0.7 mm it is lower, and for 1.4 to 2.0 mm it is greater than was observed for MPS 25 ppm. The distribution of the fragment C_3_H_5_SO_3_^−^ is similar for MPS 25 ppm and 50 ppm and grows up to position 1.5 mm and then gradually decreases. In consequence to the forward scan in the position from 0.0–0.7 mm, the ratio of CH_2_SO_3_^−^/C_3_H_5_SO_3_^−^ is significantly higher for MPS 25 ppm than for MPS 50 ppm. For position 0.8–1.6 mm the ratio is very similar, while for the range from 1.7–2.0 mm it is slightly higher for MPS 25 ppm. The ratio of total chloride/total thiols for MPS 25 ppm and MPS 50 ppm is only very similar for the range 0.0–0.5 mm and is only slightly higher for MPS 25 ppm. Moreover, the intensity distribution of C_3_H_5_SO_3_^−^ positively correlates with chloride ions demonstrating a significant increase from 0.9 to 1.3 mm while CH_2_SO_3_ in this region significantly decreases.

It is very interesting that distribution of CuS^−^ is very similar to CH_2_SO_3_^−^ while different from C_3_H_5_SO_3_^−^ for MPS 25 ppm and MPS 50 ppm. In the previous considerations (the dip-coating paragraph) we considered that the CuS^−^ fragment corresponds to the Cu–S bond. Since distribution of CuS^−^ is very similar to CH_2_SO_3_^−^, it means that the CH_2_SO_3_^−^ fragment is yielded from thiolate molecules that are in close proximity to the copper surface while the C_3_H_5_SO_3_^−^ fragment that is yielded from the thiolate consists of a thiolate end located a greater distance from the copper.

Recently, it was shown that a lower ratio of Cl/MPS on the copper surface leads to a longer Cu–Cl distance [20]. Distribution of intensity of CuS^−^ shows that Cu–S distance for MPS 50 ppm is strongly dependent on the potential (rapid reduction is observed at the position 0.8 mm) when significant number of thiolate molecules in gauche conformation is reoriented into trans conformation. It seems that the reorientation gauche/trans process does not directly depend on chloride surface coverage, while at OCP and for low overpotential for reverse scan it is significantly stabilized by the greater surface coverage since we observe the minimum intensity ratio CH_2_SO_3_^−^/C_3_H_5_SO_3_^−^ in the region from 1.0–1.5 mm for the reverse scan. A similar, less distinct effect can also be observed for MPS 25 ppm. A significantly higher gauche/trans ratio (CH_2_SO_3_^−^/C_3_H_5_SO_3_^−^) in the region 0.0–0.7 mm can be observed for MPS 50 ppm but under lower chloride surface coverage it determines the same accelerating effect (see CV curve, Figure 4b). This shows that the final accelerating effect (CV curve shape) is determined by the total chloride/total thiols and CH_2_SO_3_^−^/C_3_H_5_SO_3_^−^ ratios, total chloride, total thiols, CH_2_SO_3_^−^ (gauche conformation), and C_3_H_5_SO_3_^−^ (trans conformation). For the rest of the overpotential region the total exchange current density is the result of the above factors.

In the range of 0.7–1.1 mm, CV curves for MPS 25 ppm and MPS 50 ppm are completely overlapped, since we can observe a similar ratio of chloride/thiolate, and intensity of total chloride, as well as CH_2_SO_3_^−^ and C_3_H_5_SO_3_^−^.

In the light of above results, we propose the mechanism of copper electrodeposition in the presence of Cl/SPS and Cl/MPS as follows.

**Figure 11:**
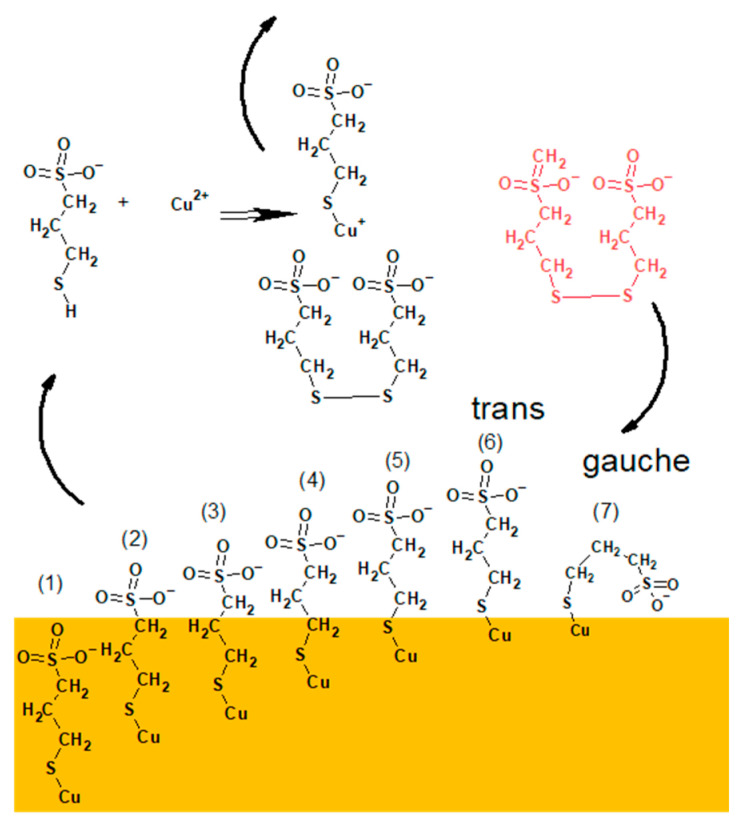
The proposed model of molecular arrangements of MPS molecules incorporated into copper deposit during copper electrodeposition from the solution contained SPS or MPS in the presence of Cl^−^ ions. Chloride ions were omitted for clarity. Desorbed MPS molecule undergoes reaction with Cu^2+^ ions. The products of complexation are accumulated into solution as a by-product. The fresh SPS molecule from solution indicated by red colour adsorbed onto copper surface.

The proposed model of distribution of SPS/MPS into and on the copper surface is shown in Figure 11.

In the first step, an SPS molecule is adsorbed on the copper surface and immediately dissociated onto two MPS units, since we did not find the existence of S–S bonds in the TOF-SIMS data (lack of fragments *m/z* = 331 and *m/z* = 371 identified in the dip-coating experiment, see paragraph). This observation is in full agreement with previous findings [12].

The accumulation of MPS units on the copper surface is dependent on the concentration of SPS in the solution and applied potential. For the lowest concentration (SPS2.5ppm), the maximum MPS coverage is observed around OCP and is positively correlated with chloride ions, while for 12.5 ppm and 25 ppm lower overpotential decreases since some MPS units are replaced by chloride ions. Adsorbed MPS molecules exist in gauche (CH_2_SO_3_^−^) and trans (C_3_H_5_SO_3_^−^) conformations. The relative amount of gauche/trans MPS units depends on the applied potential and demonstrates the highest value for SPS2.5ppm and decreases in SPS12.5ppm and SPS25ppm. The thiolate end in MPS in the gauche conformation is in closer proximity to the copper surface than in trans conformation. During the etching experiment (Section 2.2), we demonstrated that both gauche and trans forms of MPS molecules were incorporated into the copper deposit while gauche conformation was more preferentially incorporated with the chloride ions. This is an essential difference in comparison to the model presented in Figure 9 where MPS molecules were not incorporated into deposit.

Moreover, only small amounts of thiolate molecules are desorbed (Figure 11) and the greater ones are incorporated into deposit. It depends on the SPS/MPS and Cl concentration in the solution. Incorporation of thiolate into deposit may be induced by the existence of a few monolayers of thiolate on the copper surface that significantly reduce MPS desorption. The presence of a few MPS/SPS monolayers makes it difficult for closer proximity of Cu(I)MPS and SPS (the second product of reaction shown in Figure 11) to the copper surface. Instead of these SPS molecules, the SPS ones from the solution indicated by red colour in Figure 11 are able to efficiently adsorb on the copper surface.

The greater density of MPS molecules in the first monolayer and the existence of subsequent monolayers (amounts) of SPS are absolutely necessary for inducing the closer proximity of SO_3_^−^Cu^2+^. This is determined by the incorporation of MPS molecules into the copper deposit as shown in Figure 11. The number of direct interactions between SO_3_^−^ and Cu^2+^ reduce the energy of solvation of Cu^2+^ that accelerates the Cu^2+^ reduction to Cu^+^ and transfers the Cu^+^ to the Cl adlayer [8]. In consequence, the accelerating abilities are improved. The direct interactions between sodium cations and chloride ions on silver substrate were also proven by SERS studies [41].

### 2.6. Copper Electrodeposition from Base Solution with Additives: MPS or SPS without Cl

**Figure 12:**
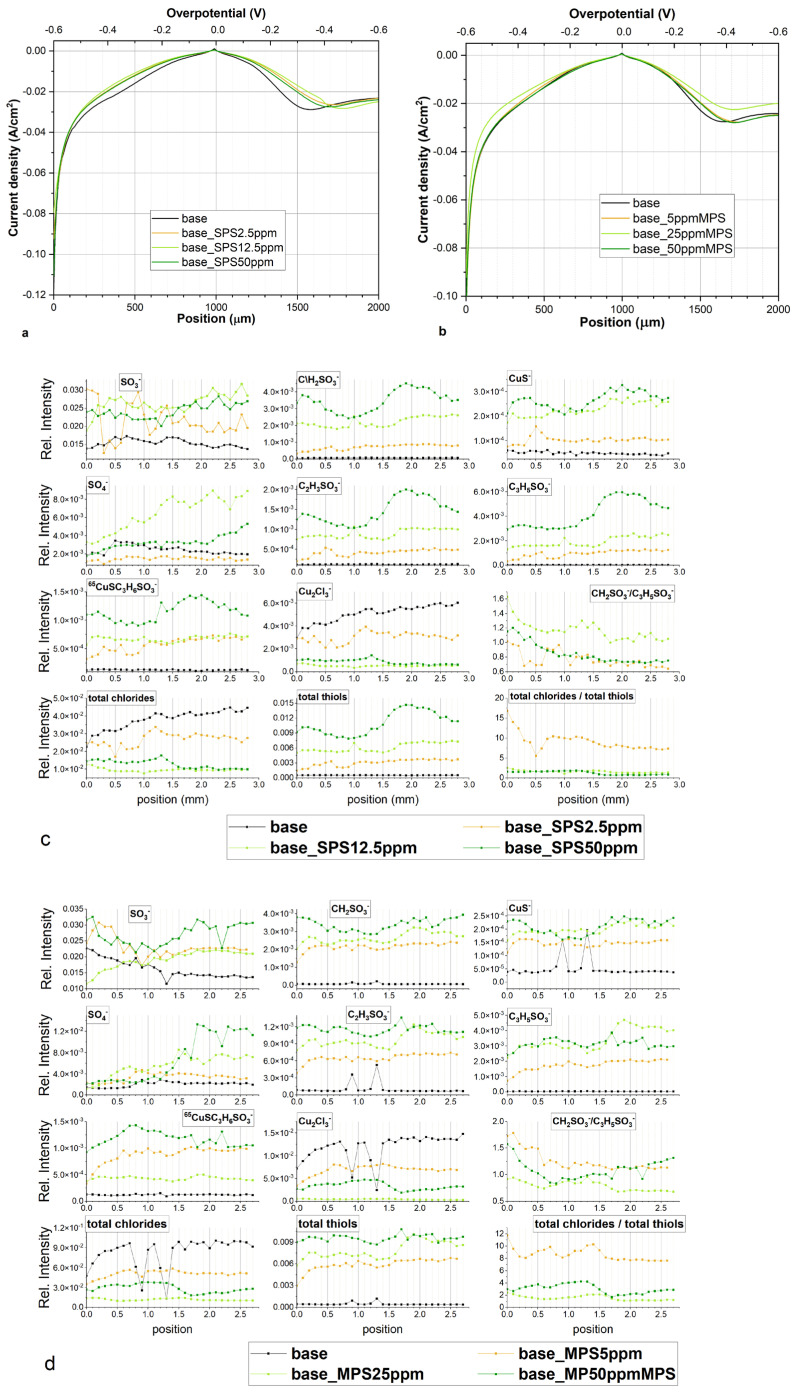
(**a**) Cyclic voltammetry curves recorded for base electrolyte, after addition of SPS at the concentrations 2.5, 12.5, and 50 ppm. (**b**) Cyclic voltammetry curves recorded for base electrolyte, after addition of MPS at the concentrations 5, 25, and 50 ppm. (**c**) Distribution of intensity of the fragments: SO_3_^−^, CH_2_SO_3_^−^, CuS^−^, SO_4_^−^, C_2_H_3_SO_3_^−^, C_3_H_5_SO_3_^−^, CuSC_3_H_6_SO_3_^−^, Cu_2_Cl_3_^−^, the ratio of CH_2_SO_3_^−^/C_3_H_5_SO_3_^−^, total chlorides total thiols, and the ratio of total chlorides/total thiols, along with the wire position for base electrolyte, base + 2.5 ppm SPS, base + 12.5 ppm SPS and base + 25 ppm SPS. The wire position corresponds to the applied overpotential during CV experiment. (**d**) Distribution of intensity of the fragments: SO_3_^−^, CH_2_SO_3_^−^, CuS^−^, SO_4_^−^, C_2_H_3_SO_3_^−^, C_3_H_5_SO_3_^−^, CuSC_3_H_6_SO_3_^−^, Cu_2_Cl_3_^−^, the ratio of CH_2_SO_3_^−^/C_3_H_5_SO_3_^−^, total chlorides total thiols and the ratio of total chlorides/total thiols along with the wire position for base electrolyte, base + 5 ppm MPS, base + 25 ppm MPS and base+ 50 ppm SPS. The wire position corresponds to the applied overpotential during CV experiment.

Cyclic voltammetry curve for the base solution and after the addition of 2.5, 12.5, and 50 ppm SPS is shown in Figure 12a. After injection, a slight suppressing ability can be observed. Accordingly, the exchange current density j_0,2_ for base and base/2.5 ppm SPS is equal to 5.21 and 2.66 mA/cm^2^, respectively. Intensity of thiolate ions: CH_2_SO_3_^−^, C_2_H_3_SO_3_^−^, C_3_H_5_SO_3_^−^, CuS^−^, and CuSC_3_H_6_SO_3_^−^ increases proportionally to the concentration of SPS in solution (Figure 12c). Total chloride intensity decreases as the amount of thiolate increases. The fragment Cu_2_Cl_3_^−^ corresponds to the copper surface not covered by thiolate molecules as similarly observed during the dip-coating experiment (Section 2.1) and corresponded to a high intensity of CuCl_2_^−^ fragment for Samples 6, 8, 13, 14, 16. The uncovered copper surface area very easily yields CuCl_2_^−^ or Cu_2_Cl_3_^−^ fragments due to impurities from the air adsorbed on the copper. Thiolate surface coverage is rather stable over the whole overpotential range for 2.5 and 12.5 ppm of SPS while for 50 ppm SPS decreases towards OCP for the forward scan as well as for the reverse scan. The ratio CH_2_SO_3_^−^/C_3_H_5_SO_3_^−^ does not demonstrate a specific correlation to SPS concentration. Significant differences between thiolate surface coverage exhibit small suppressing abilities, meaning that accelerating abilities of SPS only occur with the existence of a chloride adlayer. Adsorbed MPS molecules on the copper surface without chloride ions block and limit the access of Cu^2+^ to the copper surface. This conclusion is in full agreement with previous studies [2,3,21].

Similarly, small suppressing abilities take place for solution containing MPS without chloride ions (Figure 12b). The exchange current density j_0,2_ for base and base/5 ppm MPS solution is equal to 4.18 and 2.15 mA/cm^2^, respectively. As observed for SPS, the intensity of total thiols is proportional to the MPS concentration (Figure 12d). The amount of thiolate molecules is greater than it was for SPS. Total of chloride ions yielded from uncovered copper surface area is also similar to that observed for SPS. Similarly, as it was for SPS, the lack of chloride ions inhibits the accelerating abilities of MPS.

## 3. Materials and Methods

Dip-coating experiments (Section 2.1) were carried out in a test-tube containing 10 mL of electrolyte. During a dip-coating experiment, 32 samples were prepared (Table 1). The nitinol wire (assigned as Sample 1, NiTi/2s) was immersed for 2 s in solution containing 0.1 M MPS/0.1 M H_2_SO_4_/0.05 M CuSO_4_ and then withdrawn at a constant speed of 1 mm/s and dried in the air at room temperature. The next sample (2, NiTi/2s rinsing) was prepared in the same way, but after deposition, it was rinsed in a 5% ethanol solution. The procedure of rinsing was described in the experimental section. Similarly, the subsequent nitinol samples were prepared in the same way, whilst the immersion time and composition of additives (MPS/SPS/Cl) were varied. The same procedure of deposition process was applied to the nitinol wires with a pre-plated copper layer.

Samples were assigned as: NiTi/2s; NiTi/2s rinsing; NiTi/60s; NiTi/60s rinsing; Cu/2s; Cu/2s rinsing; Cu/60s; and Cu/60s rinsing, depending on the method of preparation.

The base electrolyte contains sulphuric acid (99.9% pure, POCH S.A., Gliwice, Poland) and copper sulphate (99% pure, Supelco, Bellefonte, PA, USA). The following additives were added: Cl^–^ (in the form of HCl, POCH S.A., Gliwice, Poland), SPS (Raschig, Ludwigshafen, Germany), and MPS (90% technical grade, Sigma-Aldrich, St. Louis, MO, USA). For preparation of all solutions, deionized water (18 MΩ, Hydrolab, Straszyn, Poland) was applied. The composition and additive concentrations in solutions applied in dip-coating are listed in Table 2. In DC (Section 2.1) and cyclic voltammetry experiments (CV) (Section 2.2, Section 2.5 and Section 2.6), a 120 mm piece of nitinol wire (0.665 mm diameter, supplied by Euroflex GmbH, Pforzheim, Germany) was immersed into copper sulphate solution in the length of 50 mm (1 cm^2^ surface area).

In the dip-coating (DC) experiments, nitinol wire or nitinol with a pre-plated copper layer at a length of 50 mm was dipped and withdrawn at constant speed V = 800 µm/s from solution. Pre-plated copper layer with thickness ~1 µm, was electrodeposited from solution containing copper sulphate (0.225 M) and sulphuric acid (0.56 M) at constant current density −20 mA/cm^2^.

In cyclic voltammetry experiments (CV) (Section 2.2, Section 2.5 and Section 2.6) accompanied by TOF-SIMS experiments, a copper ring with phosphor as an additive (45 mm outside and 30 mm internal diameter, 1 mm thickness, supplied by Lesker, Jefferson Hills, PA, USA) was employed as counter electrodes and mounted on the bottom of the test-tube. The tube contained 250 mL of electroplating bath. The purity and manufacturer of components of electroplating bath were mentioned above while the concentration of the base solution (without additives) was as follows: CuSO_4_ × 5H_2_O—0.225 M, sulphuric acid—0.56 M. Accordingly, Cl^−^ ions, SPS, and MPS in the sodium form were added as additives in varied concentrations as listed in Table 3.

A counter-electrode was connected to a galvanostat through the use of a copper wire (diameter 1 mm) covered by a thick isolated lacquer layer, resisted for electroplating bath. As a reference electrode, a Ag/AgCl electrode was applied. A galvanostat AUTOLAB 302N (Eco Chemie, Utrecht, The Netherlands) was used for cyclic voltammetry (CV) experiments. Before the addition of sulphuric acid and additives, copper sulphate solution was thoroughly deoxygenated using ultrasonic washer under reduced pressure. Before each experiment the counter-electrode was immersed into fresh 30% HNO_3_ for 30 s, then thoroughly rinsed in deionized water and dried in the air. Subsequently, nitinol wire, which served as a working electrode, was mounted vertically in electroplating bath and withdrawn at a constant speed equal to 34 µm/s from the copper electroplating bath under electrodeposition condition. Simultaneously, the CV experiment was performed, and the applied potential was swept at constant velocity, V = 20 mV/s, in the range of overpotential between −0.6 V and OCP (forward scan to anodic direction) and from OCP to −0.6 V (reverse scan to cathodic direction), where OCP is an open circuit potential. OCP was determined through 30 s or less before the CV experiment until a steady state was reached. In this manner, during CV experiment one cyclic loop corresponds to 60 s (30 s for the forward scan in the anodic direction and 30 s for the reverse scan in the cathodic direction) and 2 mm of the nitinol wire (working electrode) length during a withdrawal. Forward scan from −0.6 V to OCP corresponds to the wire position from 0 to 1 mm while the reverse scan corresponds to the position from 1 to 2 mm, respectively. For samples that were electrodeposited from solution when prior C_Cu_^2+^ > C_MPS_ or C_SPS_, to avoid the process of salting out copper sulphate on the sample surface, a rinsing procedure was applied. More details on the rinsing method can be found elsewhere [34]. Briefly, a glass capillary with an internal diameter 2.4 mm was immersed into rinsing solution (5% ethanol) and mounted horizontally. The total volume of rinsing solution inside the capillary was equal to 8 µL (height of rinsing liquid ~1 mm) and was collected at the bottom end of capillary. The rinsing properties of 5% ethanol in deionized water and other concentration of ethanolic solutions were determined in the previous work [29]. The optimal results were received for 5% ethanolic solution that from one side effectively dissolved CuSO_4_ remains and from the other side preserved the maximum amount of PEG on the copper surface. Subsequently, a sample in a vertical position was withdrawn at constant speed V = 800 µm/s through the rinsing liquid and then immediately dried in the air. As the inspected length of sample was routinely not longer than 4 mm, the volume of rinsing liquid is sufficient for cleaning copper sulphate remained. On the other hand, reduced volume of rinsing liquid and drying step reduced the contact time of liquid with underlayed MPS/SPS film, decreasing the risk of solubility of organic film.

### 3.1. Evaluation of the Molecular Composition inside the Electrodeposited Copper Layer

In this experiment, molecular composition inside the electrodeposited copper layer was examined to evaluate the possible incorporation of thiolate and chloride molecules into a copper deposit during electrodeposition. A copper layer with a thickness of 25 µm was electrodeposited from the base electrolyte consisting of 15 ppm Cl and 50 ppm MPS at constant current density −20 mA/cm^2^. Subsequently, the copper layer was etched in 30 % of nitric acid in two steps. In the first one, 5 µm of copper layer was removed, and in the second 15 µm. After each step of etching the chemistry of copper surface was measured by means of TOF-SIMS. During removal of the first 5 µm of copper layer the ultrasonic bath was used, while etching the subsequent 15 µm of copper layer was conducted without ultrasonic bath. The use of ultrasonic bath was necessary to be sure that possibility of re-adsorption of thiolate molecules onto the copper surface during etching was completely eliminated.

### 3.2. Examination of SPS Re-Adsorption and Accelerating Abilities in the Case of Lack SPS in the Solution

To evaluate the re-adsorption and accelerating abilities of MPS molecules during electrodeposition, the nitinol wire was electroplated by applied linear sweeping potential from −0.6 V to OCP in forward scan and from OCP to −0.6 V in reverse scan at 20 mV/s scan speed, in the solutions consisting of 15 ppm Cl and 50 ppm MPS or 25 ppm SPS (samples assigned as MPS/Cl or SPS/Cl). After examination of sample surface by TOF-SIMS it was transferred to the base solution but without MPS and Cl ions. Then, the one sample was immersed for 30 s under OCP condition (sample assigned as MPS/Cl/OCP) while for other one CV experiment was conducted (assigned as MPS/Cl/base).

### 3.3. TOF-SIMS Experiments

After preparation, samples were transferred to the TOF-SIMS vacuum chamber and measured routinely not later than 2–5 h after preparation. TOF-SIMS spectra were acquired by means of the TOF-SIMS.5 instrument (ION-TOF GmbH, Münster, Germany). The primary ion source of Bi^+^ was used at 30 keV (cyclic time 100 µs, primary beam current 1.2 pA). The scanning area of the secondary ions was 50 µm × 50 µm with 128 × 128 pixels and 1 shot/pixel. All the measurements were performed under the static mode (dose no larger than 1 × 10^12^ ions/cm^2^) in a negative mode. For most of samples 30 or more points separated by 0.1 mm distance were measured. The post-processing data analysis was conducted using the SurfaceLab 6.7 software (ION-TOF) and Origin 2019 (OriginLab, Northampton, MA, USA). The negative spectra were recorded and calibrated using the positions of CH^−^, CH_2_^−^, and CH_3_^−^. Intensities were normalized to the total intensity.

## 4. Conclusions

In this work we provided a new approach to studies of surface chemistry with MPS/SPS and chloride adlayer using the TOF-SIMS technique combined with cyclic voltammetry (CV). In our dip-coating experiment (Section 2.1) we proposed the method of interpretation of TOF-SIMS spectra for resolving the most important Reactions (1)–(9) between MPS, SPS, and chloride ions on the nitinol and copper substrate. We demonstrated how to distinguish primary reactions that occurred during in situ experiments (Reactions (1)–(7)) and secondary reactions (Reactions (8)–(9)) that may have appeared during TOF-SIMS measurements. Moreover, to reproduce the reaction that can occur near the surface electrode, dip-coated layers were not rinsed. On the other hand, after rinsing the deposited layers, the possible appearance of the chemical bonds on the copper surface may be helpful in reproducing and examination of copper electrode under electrochemical conditions. Analysis of cyclic voltammetry curves (Section 2.2) demonstrates that j_0,1_ and j_0,2_ exchange current densities are varied for SPS while for MPS only j_0,2_ is varied in a function of the concentration. It was concluded that in the presence of Cl the accelerating abilities of MPS rely mainly on the ion migration reactions while in the case of SPS on the inner-sphere mechanism. Without chloride ions, both MPS and SPS do not demonstrate accelerating abilities (Section 2.6). In the etching experiment (Section 2.3) molecular composition inside of a copper electrodeposited layer was examined. It was proven that MPS and chloride ions are incorporated. This fact has a great impact on the mechanism of interaction of additives with the copper and chloride adlayer. Incorporation of MPS molecules allows for maintaining of close proximity sulfonate groups and Cu^2+^ even for MPS molecules occurring in trans conformation. After electrodeposition of the copper layer from solution containing MPS/SPS and chloride ions and transferring samples to the base solution (without additives), the accelerating abilities were lost (Section 2.4). On the other hand, under these circumstances, a stable thiolate monolayer was clearly identified. This means that incorporation of MPS molecules into copper deposit did not take place while full re-adsorption occurred. Similarly, total re-adsorption occurs under an open circuit potential condition. TOF-SIMS measurements (Section 2.5) of the copper electrodeposited layer during the CV experiment (Section 2.1) allowed for examination of MPS, SPS, and chloride surface coverage. It was shown that the ratio of Cl/thiolate exhibits a great impact on the accelerating abilities. At the greatest examined SPS concentration (25 ppm) accelerating abilities were reduced due to possible accumulation of excess Cu(I)SC_3_H_6_SO_3_ complex that play a role of a suppressant. On the other hand, at 12.5 ppm the maximum accelerating abilities were found. Furthermore, accelerating abilities of MPS were maintained for a wider range of concentrations (25 and 50 ppm). The accelerating mechanism of MPS and SPS in the presence of chloride ions relies on the ion pair interaction of sulfonate ends and Cu^2+^ that facilitates dehydration, followed by the reduction of Cu^2+^ to Cu^+^ and then transfers Cu^+^ ions to the chloride adlayer. The latter process is favourable for MPS molecules remaining in gauche conformation on the copper surface and incorporated into the copper deposit both in gauche and trans conformation. It was proposed to assign the CH_2_SO_3_^−^ fragment to the gauche conformation and C_3_H_5_SO_3_^−^ to the trans conformation. Despite the high thiolate surface coverage, MPS and SPS molecules do not demonstrate accelerating abilities without chloride ions (Section 2.6), while exhibiting rather minor suppressing potential.

## Figures and Tables

**Table 1:** The characteristic fragments identified by TOF-SIMS in the samples obtained in dip-coating experiment.

No.	Centre Mass (u)	Assignment	Abbreviation
1	80.00	SO_3_^−^	
2	94.02	CH_2_SO_3_^−^	
3	94.95	CuS^−^	
4	96.00	SO_4_^−^	
5	97.00	HSO_4_^−^	
6	107.03	C_2_H_3_SO_3_^−^	
7	121.05	C_3_H_5_SO_3_^−^	
8	154	S(CH_2_)_3_SO_3_^−^	**MPS–H**
9	155.05	SH(CH_2_)_3_SO_3_^−^	**MPS**
10	177.04	S(CH_2_)_3_SO_3_Na^−^	**NaMPS**
11	217.01	CuS(CH_2_)_3_SO_3_^−^	**CuMPS**
12	309.10	S_2_H[(CH_2_)_3_SO_3_]_2_^−^	**SPS–H**
13	331.08	S_2_[(CH_2_)_3_SO_3_]_2_Na^−^	**Na–SPS**
14	371.07	CuS_2_[(CH_2_)_3_SO_3_]_2_^−^	**Cu–SPS**

**Table 2:** The assignment of samples that were obtained by dip-coating experiment. The scheme of assignments: NiTi/2s—nitinol wire immersed for 2 s; NiTi/2s rinsing—nitinol wire immersed for 2 s and then rinsed; NiTi/60s—immersion time—60 s; Cu—nitinol wire pre-plated by copper.

	NiTi/2s	NiTi/2s Rinsing	NiTi/60s	NiTi/60s Rinsing	Cu/2s	Cu/2s Rinsing	Cu/60s	Cu/60s Rinsing
**MPS**	1	2	3	4	5	6	7	8
**SPS**	9	10	11	12	13	14	15	16
**MPS/Cl**	17	18	19	20	21	22	23	24
**SPS/Cl**	25	26	27	28	29	30	31	32

**Table 3:** The scheme of CV and TOF-SIMS experiments.

	Cl (15 ppm)	SPS	MPS	CV Experiment (Section)	TOF-SIMS
**Base**	-	-	-	2.2, 2.5, 2.6	2.2, 2.5, 2.6
**Cl**	+	-	-	2.2	2.5
**SPS**	-	+(2.5, 12.5 or 25 ppm)	-	2.6	2.6
**MPS**	-	-	+(5, 25 or 50 ppm)	2.6	2.6
**SPS/Cl**	+	+(2.5, 12.5 or 25 ppm)		2.2	2.5
**MPS/Cl**	+		+(5, 25 or 50 ppm)	2.2	2.5

## Data Availability

The TOF-SIMS data can be obtained upon reasonable request.

## Supplementary Materials

The following supporting information can be downloaded at: https://www.mdpi.com/article/10.3390/molecules27238116/s1, Figure S1: Distribution of Cu^−^ and CuCl2^−^ ions on the copper layer deposited on the silicon wafer by magnetron sputtering. Inspection area: 200umx200um. Si and Cu layers are clearly visible; Figure S2: Distribution of intensity of the fragments: Cu^−^, CH2SO3^−^, C3H5SO3^−^and CuCl2^−^ along the wire for sample deposited from the base solution contained Cl(15 ppm) and 5 ppm of MPS; Figure S3: Distribution of intensity of the fragments: Cu^−^, CH2SO3^−^, C3H5SO3^−^ and CuCl2^−^ along the wire for sample deposited from the base solution contained Cl(15ppm) and 2.5 ppm of SPS; Figure S4: Distribution of intensity of the fragments: Cu^−^ and CuCl2^−^ along the wire for sample deposited from the base solution contained Clions (15 ppm).

## Sample Availability

Samples of the compounds are not available from the authors.
