# Supplementary material for: Studies of Bis-(Sodium-Sulfopropyl)-Disulfide and 3-Mercapto-1-Propanesulfonate on/into the Copper Electrodeposited Layer by Time-of-Flight Secondary-Ion Mass Spectrometry
Source: Molecules. 2022 Nov 22;27(23):8116. doi: 10.3390/molecules27238116 (PMC9737544; doi:10.3390/molecules27238116)
Supplement: Supplementary file 1 [file molecules-27-08116-s001.zip › molecules-2029670-supplementary.pdf]

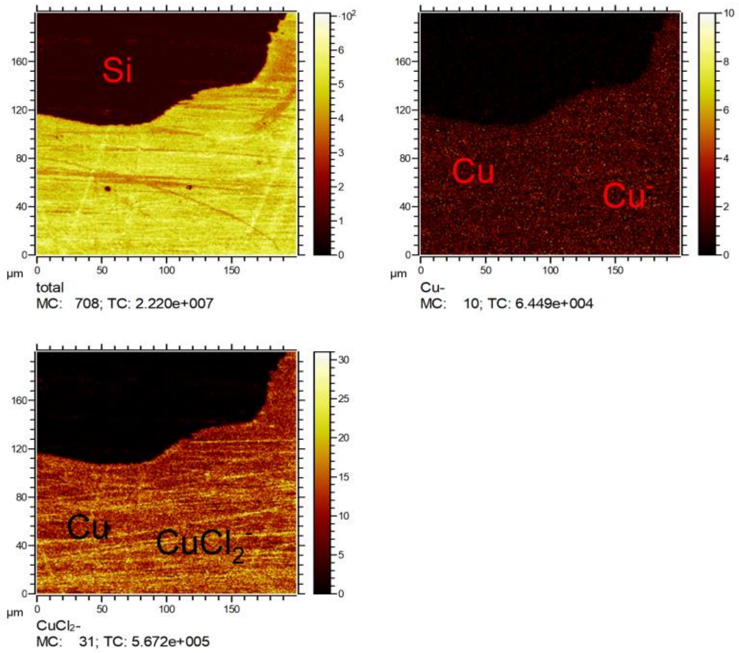

Figure S1. Distribution of Cu- and CuCl2- ions on the copper layer deposited on the silicon wafer by magnetron sputtering. Inspection area: 200umx200um. Si and Cu layers are clearly visible.

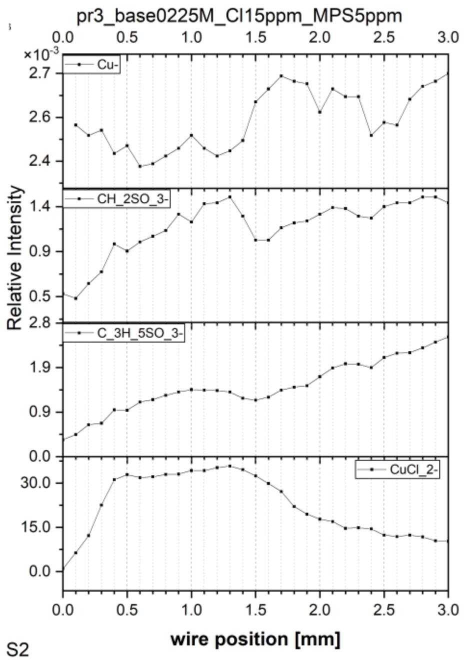

Figure S2. Distribution of intensity of the fragments: Cu-, $CH_2SO_3^-$, $C_3H_5SO_3^-$ and $CuCl_2^-$ along the wire for sample deposited from the base solution contained Cl(15ppm) and 5 ppm of MPS

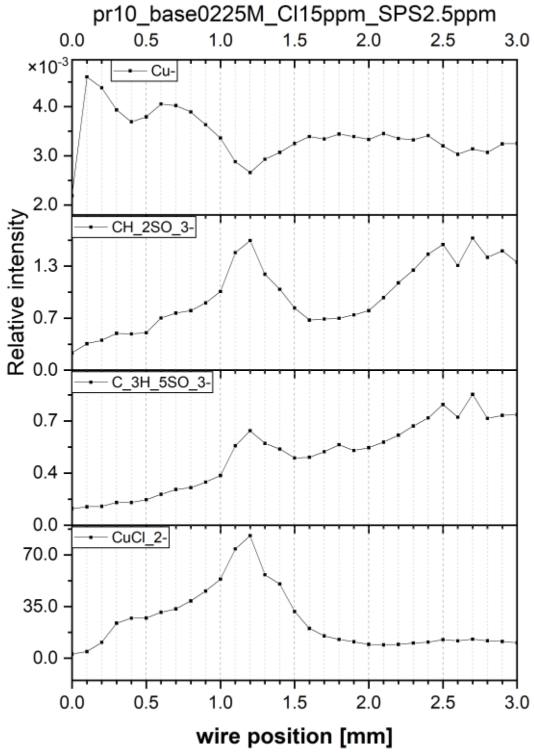

Figure S3. Distribution of intensity of the fragments: Cu-, $CH_2SO_3^-$, $C_3H_5SO_3^-$ and $CuCl_2^-$ along the wire for sample deposited from the base solution contained Cl(15ppm) and 2.5 ppm of SPS

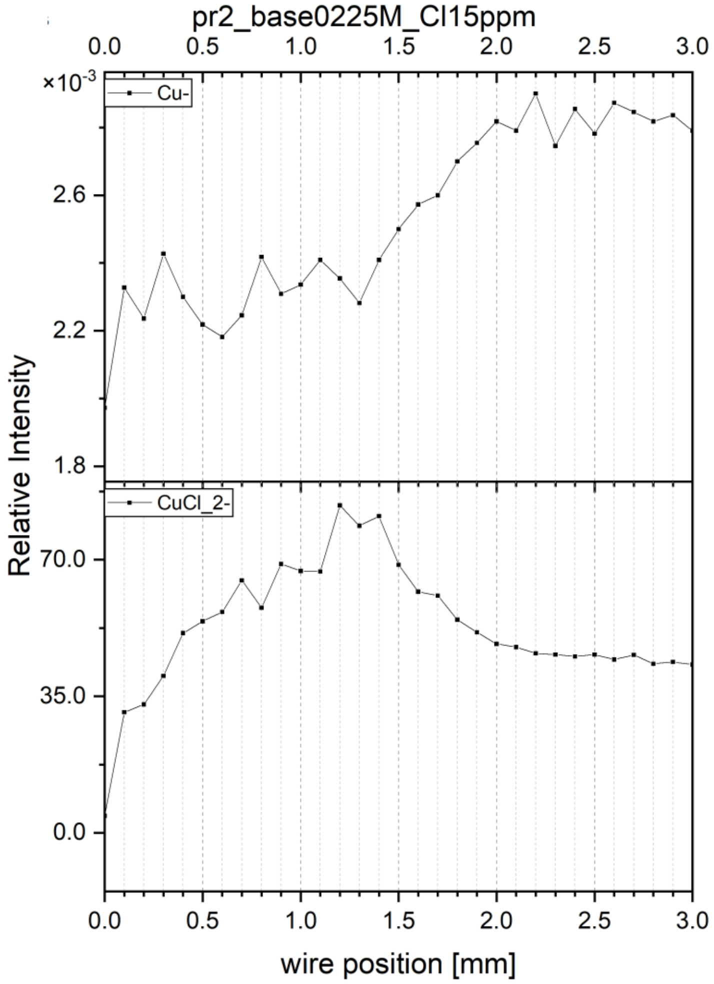

Figure S4. Distribution of intensity of the fragments: Cu- and $CuCl_2^-$ along the wire for sample deposited from the base solution contained Cl^- ions (15ppm)
